# A Bayesian state-space model using age-at-harvest data for estimating the population of black bears (*Ursus americanus*) in Wisconsin

**DOI:** 10.1038/s41598-018-30988-4

**Published:** 2018-08-20

**Authors:** Maximilian L. Allen, Andrew S. Norton, Glenn Stauffer, Nathan M. Roberts, Yanshi Luo, Qing Li, David MacFarland, Timothy R. Van Deelen

**Affiliations:** 1 Illinois Natural History Survey, University of Illinois, 1816 S. Oak Street, Champaign, IL 61820 USA; 2 Wisconsin Department of Natural Resources, 107 Sutliff Avenue, Rhinelander, WI 54501 USA; 3 Department of Forest and Wildlife Ecology, University of Wisconsin, 1630 Linden Drive, Madison, WI 53706 USA; 4 Minnesota Department of Natural Resources, 35365 800th Avenue, Madelia, MN 56062 USA; 5 Department of Statistics, University of Wisconsin, 1300 University Ave, Madison, WI 53706 USA; 6 Department of Industrial & Manufacturing Systems Engineering, Iowa State University, 3025 Black Engineering Building, Ames, IA 50011 USA

## Abstract

Population estimation is essential for the conservation and management of fish and wildlife, but accurate estimates are often difficult or expensive to obtain for cryptic species across large geographical scales. Accurate statistical models with manageable financial costs and field efforts are needed for hunted populations and using age-at-harvest data may be the most practical foundation for these models. Several rigorous statistical approaches that use age-at-harvest and other data to accurately estimate populations have recently been developed, but these are often dependent on (a) accurate prior knowledge about demographic parameters of the population, (b) auxiliary data, and (c) initial population size. We developed a two-stage state-space Bayesian model for a black bear (*Ursus americanus*) population with age-at-harvest data, but little demographic data and no auxiliary data available, to create a statewide population estimate and test the sensitivity of the model to bias in the prior distributions of parameters and initial population size. The posterior abundance estimate from our model was similar to an independent capture-recapture estimate from tetracycline sampling and the population trend was similar to the catch-per-unit-effort for the state. Our model was also robust to bias in the prior distributions for all parameters, including initial population size, except for reporting rate. Our state-space model created a precise estimate of the black bear population in Wisconsin based on age-at-harvest data and potentially improves on previous models by using little demographic data, no auxiliary data, and not being sensitive to initial population size.

## Introduction

Population estimates are essential for making decisions about management and conservation of many species^1,2^, but often are difficult or expensive to obtain across large geographical scales^2,3^. This is particularly true of mammalian carnivores^4,5^, which are cryptic and difficult to count directly^6–8^. Consequently, carnivore managers often base their population estimates on extrapolations from small data sets and adjust harvest quotas based on subjective opinion from the public and experts^9^. The importance and challenges of estimating wildlife populations has led to many different estimation methods^2,10^, and more are developed each decade (e.g.,^11–13^). For hunted populations, models using age-at-harvest data are often most practical, especially when working with a population across large scales when other methods of collecting data are difficult^2,13^. Several rigorous statistical approaches, including both frequentist and Bayesian statistics, have recently been developed that use age-at-harvest and integrate auxiliary data (usually other harvest or demographic data) to accurately estimate populations^3,11–13^. To date there has not been a model developed that creates accurate estimates without integrating auxiliary data, which makes it necessary for large field projects to collect demographic data. Bayesian state-space models may be able to accomplish this, as one of their main strengths is that they appropriately use regularization to share information across space and time in the model^11^, and may efficiently use all available data compared to other modeling approaches^13^.

Bayesian models can improve upon deterministic methods by being less reliant on prior information and allowing variation in parameters over time. Deterministic methods can sometimes be limited in accuracy^11,14^, because they rely on assumptions that demographic parameters are stable over time (e.g.,^13–15^), and can be biased when erroneous or subjective demographic parameter values are used^2,13–16^. The Bayesian state-space modelling approach allows the modeler to transparently provide biologically supported information and constraints on parameters as priors, but the models use these as a starting point and the posterior values are not dependent on the prior values provided. Bayesian state-space models are also similar to stochastic population models, in that they reduce potential bias by allowing the demographic parameters to vary over time^3,13^. Bayesian state-space models also allow for a range of information in parameters, from completely informative parameters similar to a deterministic accounting model to uninformative parameters similar to frequentist approaches, formalizing a process to transparently accommodate expert opinion when estimating wildlife populations. Drawbacks of Bayesian models is that they can be more complex and difficult to comprehend and more computationally intensive to implement than simpler models. Their implementation, however, could result in better decision-making about populations and harvest quotas, and lead to more effective monitoring and management, particularly for cryptic species.

**Figure 1:**
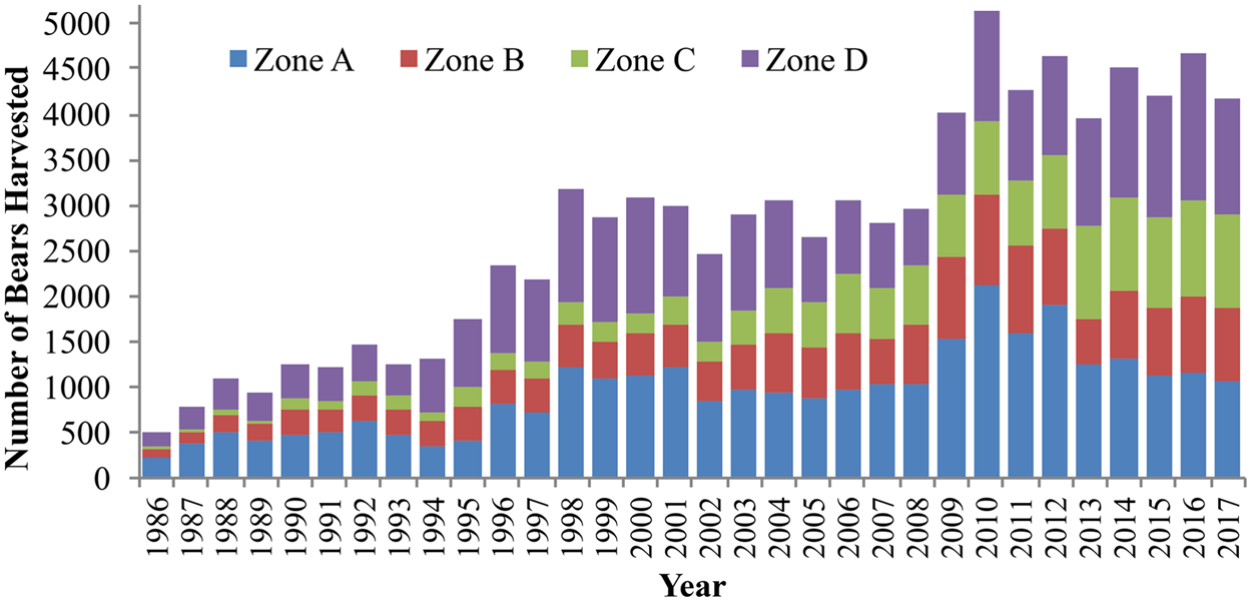
The number of harvested black bears in Wisconsin from 1971–2015, with no bear harvest in 1985. The number of harvested bears in each county is noted by a different color.

Black bears (*Ursus americanus*) are a K-selected (e.g., Pianka 1970), spatially dispersed solitary carnivore^17–20^. Black bears are a widely distributed species across North America, with many populations expanding in recent years^21^. In Wisconsin, black bears are a widespread game animal whose population and harvest have increased over the last few decadess^22,23^ (Fig. 1). Most black bears in Wisconsin are found in the northern half of the state, but the population has been expanding southward in recent years. Since 1985 the Wisconsin Department of Natural Resources (WDNR) has estimated bear populations using a deterministic accounting model^22^. However, an independent capture-recapture estimate generated from tetracycline marking found that the current model underestimated the population size by nearly 2/3^22^. This is mainly due to the inability of the deterministic model to account for variation in harvest and population demographics over time and because the model incorrectly assumes a linear relationship between independent bear abundance estimates from bait stations and population abundance^24^. Independent population estimates have allowed the WDNR to more accurately assess the black bear population in the state^22^, but these are expensive and often conducted years apart. Consequently, there is a need to update the population models in Wisconsin, as well as in many other states and jurisdictions.

K-selected species, including black bears, are susceptible to over-harvest^25^, and management agencies need to carefully track populations when setting harvest quotas and goals. Bayesian state-space models may be ideal for estimating wildlife populations^13^, but have been used less frequently by wildlife managers to date (but see^11,13^). Our goal was to create and evaluate a Bayesian state-space model using age-at-harvest data to estimate the statewide abundance of black bears in Wisconsin. Our objectives were to (1) determine reasonable prior distributions using literature review and harvest data; (2) compare abundance estimates to estimates from the capture-recapture estimates using tetracycline marking from 2011^26^ and the population trend to the trend from catch-per-unit-effort for the state; and (3) analyze the sensitivity of the state-space model’s population estimate to different specifications of the prior distributions for each demographic parameter and initial population size.

## Materials and Methods

### Study Area

**Figure 2:**
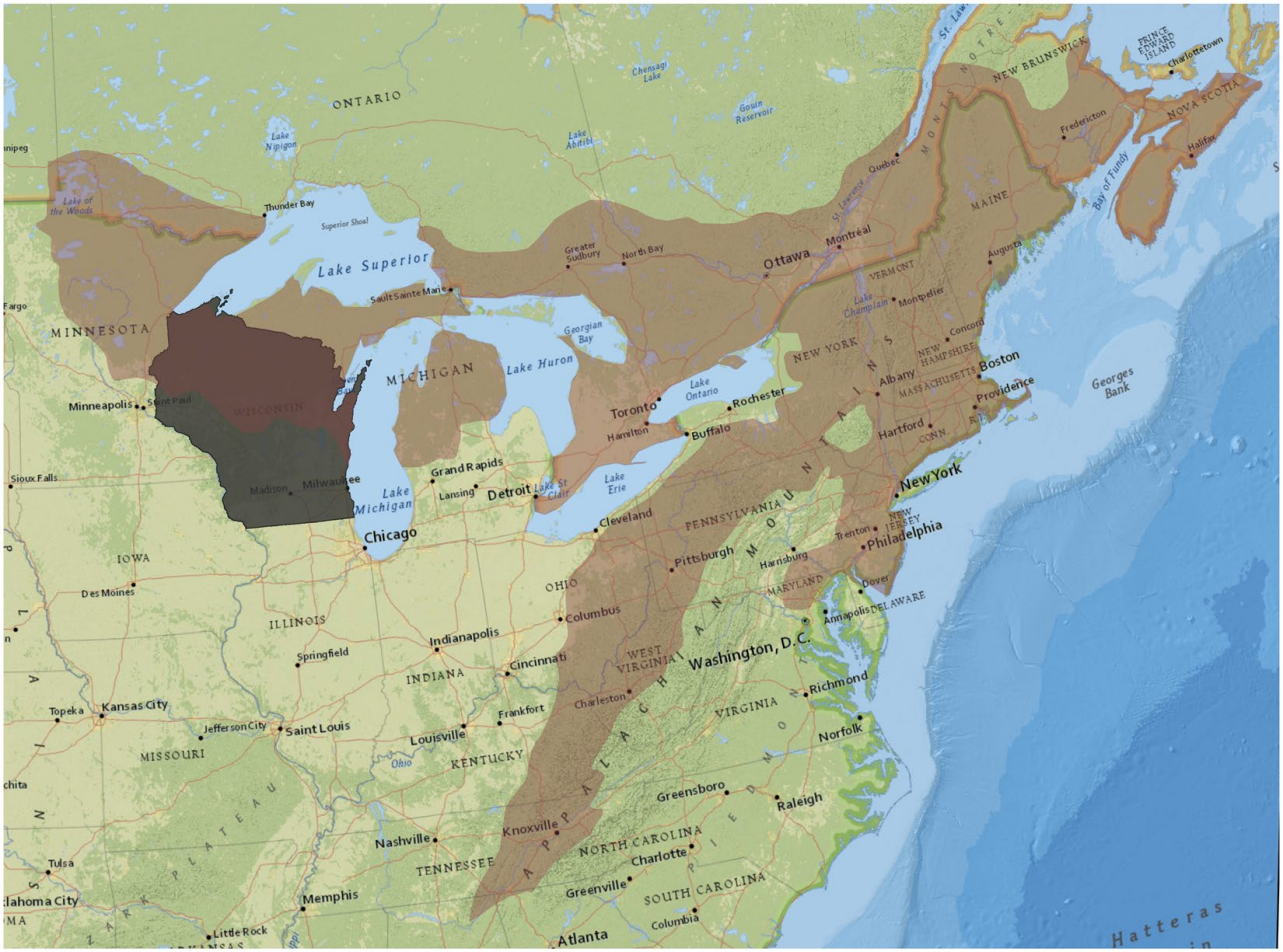
Study area of Wisconsin in gray, and quasi-study area of the northern mixed forest ecotone. We used the quasi-study area to restrict the scope of the literature review of black bear studies to develop appropriate prior distributions for demographic parameters. The figure was created with ArcGIS 10.3 (www.arcgis.com) with the National Geographic open data layer base map (http://www.esri.com/news/arcuser/0312/files/ng-basemap.pdf).

Our study focused on the black bear population for the entire state of Wisconsin (Fig. 2), where the WDNR manages bears in 4 hunting zones (Supplementary Material 1). Most of the bear population is in the northern half of Wisconsin (hunting zones A, B, and D), and each zone has unique quotas and hunting regulations^22^. Over the course of our study the bear season began on the first Wednesday after Labor Day and was open for 35 days. Our methods were carried out in accordance with approved guidelines from the WDNR and University of Wisconsin, because we only performed analyses of harvest data did not include any experimental protocols or handling of animals. All data collected by the WDNR is archived by WDNR data scientists and is fully available to the public. The data used for analyses in this manuscript are available within the manuscript and associated supplementary material.

We used reasonably informative prior distributions for the model parameters. Because information from Wisconsin for such prior distributions was sparse, we relied on studies from surrounding areas. To limit potential bias due to variation between Wisconsin and other study areas (e.g.,^27^), we defined a quasi-study area based on habitat. We used areas in the northern temperate mixed forest ecotone (Fig. 2), in an attempt to match the habitat of the three northern Wisconsin bear zones. We included all mixed deciduous, coniferous and broad-leaved forest types delineated by Bailey^28^ in ArcGIS 10.4 (ESRI, Redlands, CA) to create the quasi study area (Fig. 2). We reviewed estimates (or data when available) from all available peer-reviewed literature from within the quasi-study area to set relevant prior distributions for model parameters.

### Population Size Parameters

Our goal was to estimate the total abundance (*N*) of the black bear population in Wisconsin immediately preceding the hunting season. We denote this population size as ${N}_{a,s,y}$, where *a*, *s*, and *y*, respectively, denote age, sex, and year for the indicated population size. We initialized the model with *N*_*total*_ = 21,450 in 2009, based on estimates from the WDNR (Supplementary Material 2a). Proportions of *N*_*total*_ in each age class were approximations based on the mean proportions observed in each age class in the bear harvest over the previous 30 years. As with most other population models, we assumed that harvest was proportional to the population for each age class. We therefore visually assessed the harvest proportion by age class over 30 years and found similar proportions despite increases in harvest, and therefore considered the proportions accurate enough for use in a Bayesian modelling framework, which uses the priors to inform the posteriors of the model.

### Harvest Data

Our harvest data included:

***O*** = observed total harvest by year (***y***), which we assumed to be a complete count of legal harvest.

***C*** = number of harvested bears with known age (***a***) and sex (***s***). In the model, ***a*** is written as 10 age classes (1.5-year-olds, 2.5-year-olds, …, 9.5-year-olds, >10.5-year-olds), excluding cubs (0.5-year-olds) that cannot be legally harvested.

We used 8 years (2009–2016) of black bear harvest data from Wisconsin. Since 1973, the WDNR has required bear hunters to register all harvested bears. We used these data to account for the total annual observed harvest. Sex of animals was recorded, and a tooth was extracted from each animal and submitted to Matson’s Lab (Milltown, MT, USA) for aging through analysis of cementum annuli^29^. In a small proportion of bears, accurate aging was not possible. Thus

$${{\rm{O}}}_{{\rm{y}}} > {\sum }^{}{{\rm{C}}}_{{\rm{a}},{\rm{s}},{\rm{y}}}.$$

### Recruitment Parameters

Our recruitment variables included:

***LS***_***a***_ = age-specific mean litter size of black bears,

***PR***_***a***_ = age-specific pregnancy rate (annual probability of giving birth), based on the proportion of bears that have first litters at given ages, then the interbirth interval for subsequent litters,

***SP***_***s***_ = proportions of newborn cubs that are female and male.

We reviewed the literature on cub survival to specify prior distributions for:

***CubSa*** = Cub survival from birth to the beginning of the first harvest season,

***CubSb*** = Cub survival from the beginning of the first harvest season to the beginning of the second harvest season.

Because it is illegal to harvest black bear cubs, bears do not enter the harvest model until they reach 1.5 years of age (immediately preceding the harvest season). Age-specific fecundity values (as number of 1.5-year-olds entering the model, per female) were calculated as:

$$Fe{c}_{a}=L{S}_{a}\times P{R}_{a}\times CubSa\times CubSb$$

and multiplied by the number of females in each age class of the previous year to determine the number of 1.5-year-olds entering the population and by *SP*_*s*_ to determine the proportion by sex. We back-calculated the number of 0.5-year-olds in the population model as:

$${N}_{0.5,s,y-1}={N}_{1.5,s,y}/CubSb$$

Based on our literature review, we assumed that 0.5- and 1.5-year-old bears did not produce any cubs, but that a small proportion of the 2.5-year-old bears would have given birth at 2 years of age, and we therefore defined 4 fecundity age groups (2.5, 3.5, 4.5, and 5.5+ year-olds). These age groups are aggregated differently from the groups defined for abundance, but the subscript *a* denotes actual age (except for the absorbing terminal age of the 10.5+ -year-old age class), so its use is consistent.

To specify prior distributions for ***LS***_***a***_ we reviewed literature from our quasi-study area (Table 1). Because of substantial differences in litter sizes between first and subsequent litters we used data only from studies from which we could determine values for first and/or subsequent litters, and then used these studies to parameterize the prior distributions (Table 2).

**Table 1:** Review of mean litter sizes from studies in the northern hardwood ecotone, listed in order of sample size. Litter sizes are split into all litter sizes, and those for first litters and later litters.

Source	State/Province	All Litters			First Litter			Later Litters		
		n	LS	Range	n	LS	Range	n	LS	Range
^46^	Virginia	*n/a*	*n/a*	1–4	*n/a*	*n/a*	*n/a*	*n/a*	*n/a*	*n/a*
^47^	Maine	259	2.4	1–4	69	2.0	1–4	190	2.5	1–4
^19^	Minnesota	101	2.5	1–5	29	2.0	*n/a*	72	2.7	*n/a*
^48^	Massachusetts	86	2.3	1–4	20	1.6	1–3	66	2.6	1–4
^17^	Minnesota^a^	52	2.4	1–3	17	2.1	1–3	35	2.5	1–3
^40^	Tennessee	45	2.6	1–4	*n/a*	*n/a*	*n/a*	*n/a*	*n/a*	*n/a*
^49^	Massachusetts	27	2.4	1–4	*n/a*	*n/a*	*n/a*	*n/a*	*n/a*	*n/a*
^50^	Virginia	26	2.3	1–4	*n/a*	*n/a*	*n/a*	*n/a*	*n/a*	*n/a*
^41^	Ontario	18	2.5	1–4	*n/a*	*n/a*	*n/a*	*n/a*	*n/a*	*n/a*
^17^	Minnesota^b^	18	3.0	1–4	8	2.5	1–3	10	3.4	3–4
^42^	all litter sizes, and those for first litters and later	15	2.5	2–4	*n/a*	*n/a*	*n/a*	*n/a*	*n/a*	*n/a*
^51^	Virginia and North Carolina	7	2.3	1–3	*n/a*	*n/a*	*n/a*	*n/a*	*n/a*	*n/a*

^a^In a natural system.

^b^In a system with access to garbage.

We provide the sample size (n), mean litter size (LS), and the range of litter sizes. Cases where data is not available are marked as not available (*n/a*).

**Table 2:** Prior distributions and hyperparameters in our statewide Bayesian state-space model using age-at-harvest data, split into recruitment and survival parameters.

Recruitment Parameters				
Variable	Parameter	Mean	Distribution	
*LS-a*	Litter Size 2.5-year-olds	2.00	Gamma (20,10)	
*LS-b*	Litter Size 3.5-year-olds	2.00	Gamma (20,10)	
*LS-c*	Litter Size 4.5-year-olds	2.00	Gamma (20,10)	
*LS-d*	Litter Size 5.5+ year-olds	2.74	Gamma (16.4,6)	
*PR-a*	Pregnancy Rate 2.5-year-olds	0.003	Beta (2.61,1000)	
*PR-b*	Pregnancy Rate 3.5-year-olds	0.25	Beta (34,100)	
*PR-c*	Pregnancy Rate 4.5-year-olds	0.53	Beta (54,48)	
*PR-d*	Pregnancy Rate 5.5+ year-olds	0.48	Beta (47,50)	
*SP*	Sex Proportion (female)	0.46	Beta (426, 500)	
**Survival Parameters**				
**Variable**	**Parameter**	**Mean**	**Long-Term Precision**	**Annual Precision**
*HSm*	Male Harvest Survival	0.77	3	Gamma (20,0.5)
*HSf*	Female Harvest Survival	0.85	3	Gamma (20,0.5)
*NS*	Non-harvest Survival	0.95	4	Gamma (20,0.5)
*CubSa*	Cub Survival years 0.0–0.5	0.84	4	n/a
*CubSb*	Cub Survival years 0.5–1.5	0.71	4	n/a
*Rep*	Recovery Rate	0.98	2	n/a

We include the variable, parameter description (for gamma distributions these are the shape and rate), mean and distribution used. For survival prior distributions the means are given at the real parameter scale and long-term and annuals precisions (1/variance) are at the link scale (loglog).

To specify prior distributions for ***PR***_***a***_ we used birth data from Wisconsin black bears determined through cementum annuli techniques^30^. To determine the age-specific probability of having a first litter, we used data from 1989 to 2008, and calculated the annual mean proportion of bears giving birth for the first time for each age class. We also used the interbirth interval values provided by the authors^30^, used these hyperparameter values for the prior distributions (Table 2).

To specify prior distributions for ***SP***_***s***_ we reviewed literature from our quasi-study area, but found only one study^19^ with robust sample sizes (e.g., n > 20) and therefore used the values from that study as our hyperparameters for the prior distributions (Table 2).

For cub survival data (***CubSa*** and ***CubSb***; Table 3) we reviewed literature from our quasi-study area to determine the prior distribution and hyperparameters (Table 2).

**Table 3:** Review of black bear survival from studies in the northern hardwood ecotone, listed in order of sample size.

Source	State/Province	Annual			Harvest Season			Non-Harvest Season		
		Survival	n	Range	Survival	n	Range	Survival	n	Range
**Male**										
^38^	North Carolina	n/a	n/a	n/a	0.69	16	0.27–0.89	1.00	16	1.00–1.00
^52^	Pennsylvania	n/a	n/a	n/a	0.78	4324	n/a	n/a	n/a	n/a
^51^	North Carolina and Virginia	0.59	n/a^+^	n/a	0.71	n/a^+^	n/a	0.84	n/a^+^	n/a
^50^	Virginia	0.59	22	0.38–0.76	n/a	n/a	n/a	n/a	n/a	n/a
^31^	Ontario	0.83	n/a	n/a	n/a	n/a	n/a	n/a	n/a	n/a
^39^	Virginia	0.49	65	0.15–0.88	0.53	65	0.16–0.88	1.00	31	1.00–1.00
^53^	Minnesota	n/a	n/a	n/a	0.80	n/a	0.75–0.83	n/a	n/a	n/a
^54^	North Carolina^*^	0.69	72	0.60–0.75	n/a	n/a	n/a	n/a	n/a	n/a
**Female**										
^38^	North Carolina	n/a	n/a	n/a	0.71	35	0.53–0.82	1.00	35	1.00–1.00
^55^	North Carolina	0.70	101	0.59–0.83	n/a	n/a	n/a	n/a	n/a	n/a
^52^	Pennsylvania	n/a	n/a	n/a	0.84	2685	n/a	n/a	n/a	n/a
^51^	North Carolina and Virginia	0.87	n/a^+^	n/a	0.90	n/a^+^	n/a	0.96	n/a^+^	n/a
^50^	Virginia	0.93	24	0.77–0.99	n/a	n/a	n/a	n/a	n/a	n/a
^31^	Ontario	n/a	n/a	n/a	n/a	n/a	n/a	0.84	n/a	0.82–0.85
^39^	Virginia	0.90	76	0.52–0.99	0.91	76	0.51–0.99	1.00	56	1.00–1.00
^53^	Minnesota	n/a	n/a	n/a	0.87	n/a	0.86–0.90	n/a	n/a	n/a
^54^	North Carolina^*^	0.69	72	0.60–0.75	n/a	n/a	n/a	n/a	n/a	n/a

Survival values are split by sex with values for annual survival, harvest season survival, and for non-harvest season survival, when available. We list the sample size (n), the mean survival estimate, and the range of survival values provided. Cases where data were not available are marked as not available (n/a).

^*^Reported for males and females combined. ^+^51 bears in total.

### Survival Parameters

Our survival variables included:

***HS***_***a,s,y***_ = age-, sex-, and year-specific survival during harvest season,

***NS***_***y***_ = age- and sex-specific survival outside of harvest season,

***Rep***_***s,a***_ = sex- and age-specific recovery rate of bears during hunting season (percentage of hunting season mortality related to legal, reported harvest),

***LHR***_***a***_ = age-specific offset term for complementary log-log survival during the hunting season.

Harvest rate, noted as:

***HR***_***a,s,y***_ = age-, sex-, and year-specific harvest rate,

was then a latent variable calculated annually as

$$H{R}_{a,s,y}=(1-H{S}_{a,s,y})\times Re{p}_{a,s}$$

Age- and sex-specific survival was then calculated annually as:

$${S}_{a,s,y}=H{S}_{a,s,y}\,\times N{S}_{y}$$

For adult survival parameters (***HS***_***s***_, ***NS***, and ***Rep***_***a,s***_) we reviewed literature from our quasi-study area (Table 3), to determine the prior distributions and hyperparameter values (Table 2). We based the distribution and mean for reporting rates on a pair of studies from Ontario^31^ and Minnesota^19^. Because Wisconsin requires registration for every animal harvested, the reporting rate in Wisconsin is thought to be nearly universal and noticeably higher than reporting rates in Minnesota where registration is voluntary, and consequently we based the mean on the study from Ontario (Table 2), where every hunter was sought out^31^.

### Parameter Summary

In summary, our modeled population parameters are: ***N***_***y***_, ***LS***_***a***_, ***PR***_***a***_, ***SR***_***s***_, ***HS***_***a,s,y***_, ***NS***_***y***_, ***CubSa***, ***CubSb***, and ***Rep***_***a,s***_, and the harvest data are *O*_*s,y*_ and *C*_*a,s,y*_. All other parameters (latent parameters) were derived from the basic parameters (e.g., ***HR***_***a,s,y***_). Regularization of parameter estimates was achieved by construction of informative prior distributions for each modeled parameter, based on information about black bear ecology.

### Modeling Framework

**Figure 3:**
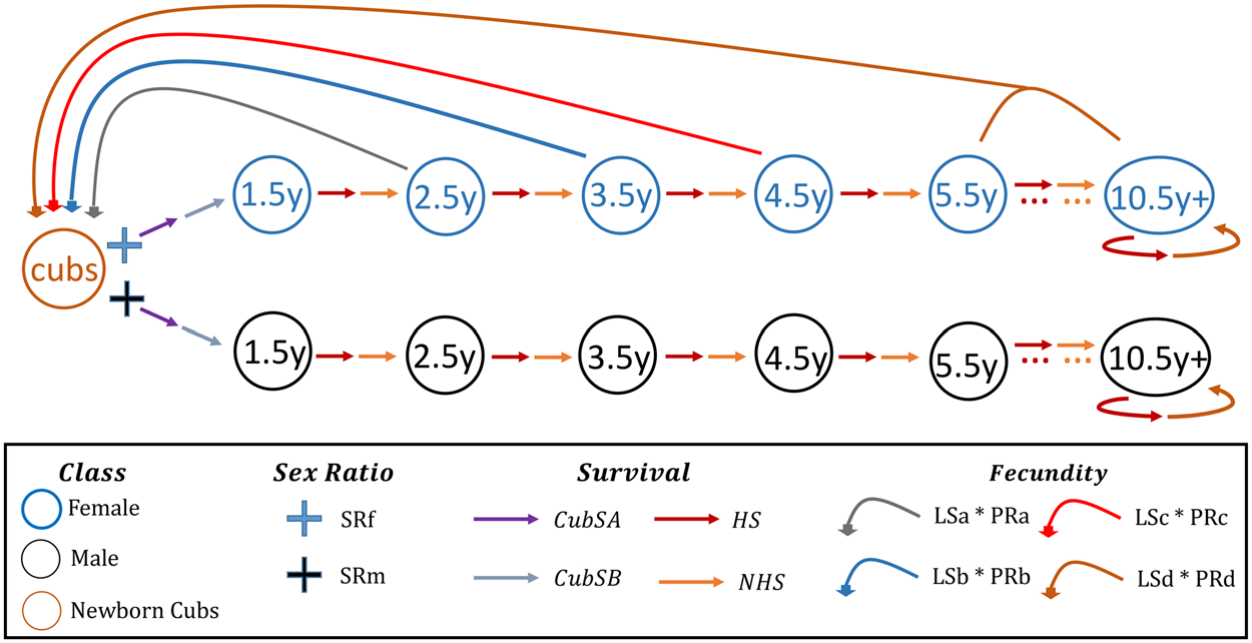
Life cycle diagram of black bears used to construct the stage-structured population matrix.

Our state-space model consisted of two process models whose likelihoods were jointly modeled^13,32^. The population process model (Fig. 3) was based on the unobserved/latent population state process (that progresses from the initial state density to sub-state transitional densities [hunting season, non-hunting season, recruitment]), and the observation state process was based on observed harvest data^13,33^. We used Markov Chain Monte Carlo (MCMC) methods to approximate posterior distributions^13^ and based our inference on posterior summaries of the MCMC samples.

Our population process model was constructed as a two-sex, ten-stage population projection matrix^10^. The age distribution for the starting population in year 1 was specified by our prior distribution. In subsequent years, abundances for age classes ≤2 were derived as

$${N}_{a,s,y}={N}_{a-1,s,y-1}\times {S}_{a-1,s,y-1}.$$

The abundance of the terminal age class in years 2 − Y was

$${N}_{A,s,y}={N}_{A-1,s,y-1}\times {S}_{A-1,s,y-1}+{N}_{A,s,y-1}\times {S}_{A,s,y-1},$$

because this was an absorbing age class. Abundance in the first age class in year *y* = 2 to Y was dependent on survival of cubs produced in year *y* − 2, and was derived as:

$${N}_{1,s,y}=S{P}_{s}\times CubSa\times CubSb\times \sum _{a=1}^{A}{N}_{a,1,y-2}\times Fe{c}_{a}$$

Because *N*_*y-2*_ was not defined when *y* = 2, we made the necessary simplifying assumption that ${N}_{a,1,y-2}={N}_{a,1,y-1}$ when *y* = 2.

Our observed-harvest data model consisted of two parts: total observed harvest (***O***) and harvested bears that have been aged and sexed (***C***). This is necessary because only a subset of the legal harvest is aged and sexed, due to broken teeth, lost samples, or other problems. Because the harvest likelihood was constructed across all age groups each year, variation will only include sampling variation^13^.

### Statewide State-Space Model

We created a ‘statewide’ state-space population model, to estimate the black bear population in the entire state of Wisconsin using actual harvest data from 2009–2016 and our prior distributions (Table 2). We fit our models in Program R^34^ using *JAGS*^35^ and the R package *rjags*^36^ (full code available in Supplementary Material 3). We ran 220,000 iterations with 3 chains, a burn-in of 20,000, and a thinning rate of 4. We visually assessed the convergence and mixing of the chains, and used Gelman-Rubin statistics to determine convergence^37^. We visually compared the posterior abundance prediction for 2011 with WDNR capture-recapture estimates based on tetracycline marking from 2011^26^. We also compared the posterior abundance trend, and the WDNR abundance trend from the 2017 model (Supplementary Material 2b), to the trend of catch-per-unit-effort (CPUE, calculated as annual harvest divided by annual hunting permits issued) for Wisconsin using linear regressions.

### Assessing Sensitivity of State-Space Model Parameters

We essentially used only harvest data from Wisconsin, although independent auxiliary data can be used to increase the precision of parameters in state-space models when needed^13^. To understand how the hyperparameter values of our prior distributions affected the accuracy of state-space model performance, we compared the results of our statewide state-space model to models run with bias in individual parameters. We considered 10% positive and negative biases for the mean and variation of the prior distributions for 9 parameters, totaling 18 different scenarios (Table 4). In each of the 18 models for the sensitivity analyses, the hyperparameter values for each parameter were exactly the same as our statewide model except for the parameter being tested.

**Table 4:** Parameters tested for sensitivity to prior distributions, with resulting percent relative change (PRC) and error measurements as coefficient of variation (CV) in the Bayesian state-space model.

Variable	Description	PRC	CV
LS − 10%	10% Underestimate of litter size	−0.68	0.68
LS + 10%	10% Overestimate of litter size	1.09	1.09
PR − 10%	10% Underestimate of pregnancy rate	−0.98	0.98
PR + 10%	10% Overestimate of pregnancy rate	1.25	1.25
HSm − 10%	10% Underestimate of male harvest season survival	−0.04	0.04
HSm + 10%	10% Overestimate of male harvest season survival	0.02	0.02
HSf − 10%	10% Underestimate of female harvest season survival	−0.43	0.43
HSf + 10%	10% Overestimate of female harvest season survival	0.93	0.92
NHS − 10%	10% Underestimate of non-harvest season survival	1.64	1.85
NHS + 10%	10% Overestimate of non-harvest season survival	N/A	N/A
Rep − 10%	10% Underestimate of reporting rate	7.33	7.26
Rep + 10%	10% Overestimate of reporting rate	N/A	N/A
CubSa − 10%	10% Underestimate of cub season a survival	−0.44	0.43
CubSa + 10%	10% Overestimate of cub season a survival	1.30	1.30
CubSb − 10%	10% Underestimate of cub season b survival	1.46	1.48
CubSb + 10%	10% Overestimate of cub season b survival	−1.49	1.49
N − 10%	10% Underestimate of starting population	−1.81	1.84
N + 10%	10% Overestimate of starting population	1.98	2.02

As with the statewide population model, we ran the models from the sensitivity analyses using 220,000 iterations in 3 chains, with a burn-in of 20,000 and a thinning rate of 4. We used Gelman-Rubin statistics to determine convergence^37^, where we considered any values < 1.1 to indicate convergence.

To evaluate the sensitivity of the state-space model to each scenario we calculated percent relative change (PRC) in population as:

$$PRC=\frac{\sum _{y=1}^{Y}(\frac{{\underline{\hat{{\bf{N}}}}}_{y}-{\underline{{\bf{N}}}}_{y}}{{\underline{{\bf{N}}}}_{y}})}{Y}\times 100,$$

and the coefficient of variation (CV) as:

$${{\rm{CV}}}_{pop}=\frac{\sqrt{\frac{\sum _{y=1}^{Y}{({\underline{\hat{{\bf{N}}}}}_{y}-{\underline{{\bf{N}}}}_{y})}^{2}}{Y}}}{\overline{\underline{{\bf{N}}}}}\times 100$$

for comparison between models, where $\hat{N}$ is an abundance estimate from the sensitivity model and N is an abundance estimates from our statewide population model.

## Results

### Statewide Population Model

We used a Bayesian state-space model to estimate the statewide black bear population in Wisconsin using harvest data from 2009–2016. The observed mean harvest (***O***) was 4425 (+/− 140 SE) bears and ranged from 3952 to 5133 bears (Supplementary Material 4). Bears with known age and sex (***C***) comprised, on average, 85.9% of the harvest (Supplementary Material 4).

**Figure 4:**
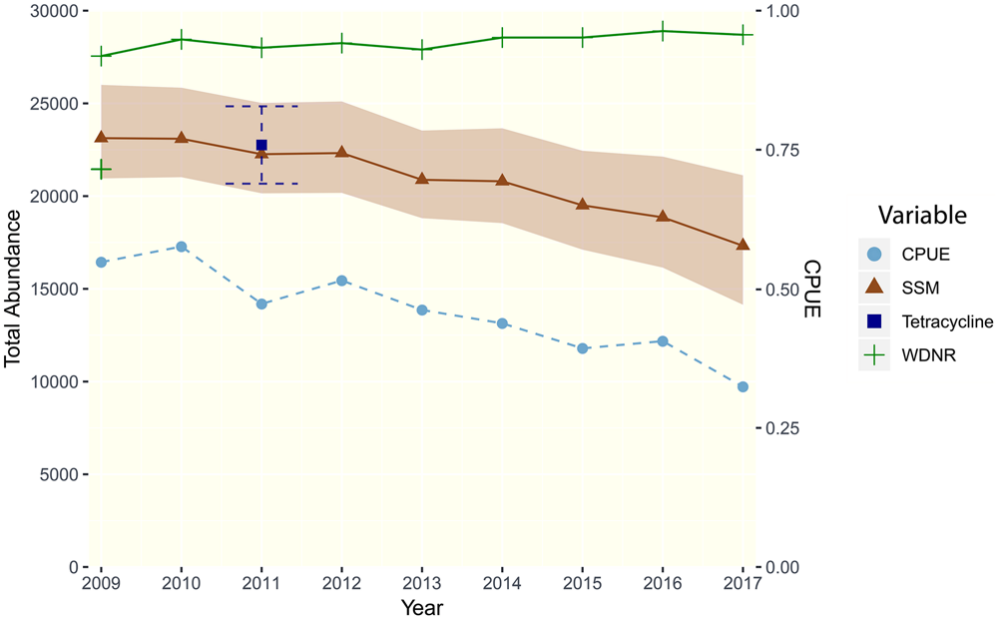
A comparison of our statewide population estimates and 95% credible intervals from the Bayesian state-space model (SSM, in brown) for Wisconsin (2009 to 2017) and the 2017 WDNR population estimate trend (in green). Also shown for comparison are the WNDR population estimate from 2009 (the initial population size for our SSM), the statewide trend in catch-per-unit-effort (CPUE, on the secondary y-axis in light blue), and the independent capture-recapture population estimate (for bears 1.5+) from tetracycline marking in 2011 with 95% confidence intervals (in dark blue).

The statewide population estimates indicated a decreasing trend in the black bear population from 2009 to 2017 (Fig. 4). The annual variation and 95% credible intervals were similar, but increased slightly in the final two years of estimation (Fig. 4). The population abundance estimate for 2011 was visually similar to the independent tetracycline estimate for 2011 (Fig. 4). The population trend estimate had a significant and strong correlation with CPUE (df = 8, R^2^ = 0.93, p < 0.0001), while the 2017 population trend from WDNR model had a non-significant correlation with CPUE (df = 8, R^2^ = 0.36, p = 0.09).

The posterior distributions and means did not differ greatly from the prior distributions and means for litter sizes (Supplementary Material 5a), pregnancy rates (Supplementary Material 5b), and sex proportion of cubs (Supplementary Material 5c). Conversely, posterior distributions for harvest season survival for each sex and year (Supplementary Material 5d) were considerably more informative than the prior distributions. Compared to the prior distributions the means of the posterior distributions for harvest survival were generally slightly lower for females and were lower in all cases for males (Supplementary Material 5d). Harvest survival for younger age classes (1.5, 2.5, and 3.5 year-olds) was lower than for older age classes, and varied among years with 2011 and 2016 having the lowest survival estimates (Supplementary Material 5). The means of the posterior distributions for non-harvest season survival for each year was higher than the means of our prior distributions, but precision did not greatly improve (Supplementary Material 5e). Similarly, the means of the posterior distribution for cub survival for both periods were slightly greater than the means of the prior distribution, and precision improved only slightly (Supplementary Material 5f). The posterior distribution for the reporting rate were more informative than the prior distribution for females, but for males the posterior distribution had slightly greater variance than the prior distribution (Supplementary Material 5g). The means of the posterior distribution for the initial population size were generally slightly lower than the means of the prior distribution for males, and generally slightly higher for females (Supplementary Material 5h).

### Sensitivity of Statewide Population Model

Based on the PRC values, our population model estimates were most sensitive to potential bias in the reporting rates, with a 10% underestimate of the reporting rate led to a PRC of 7.33 (CV = 7.26). The model was robust to potential bias in all other parameters, which had PRCs of <2.00 (Table 4). A posthoc test of 50% bias in the initial population resulted in PRCs of −7.60 (CV = 7.66) for an underestimate and 11.88 (CV = 12.03) for an overestimate.

In each sensitivity test the models closely tracked the slightly decreasing trend and abundance estimates of our statewide state-space model. The potential bias of each variable also resulted in the expected population effects (increase or decrease of estimate), except in the cases of non-harvest season survival and cub survival for period B. In these cases, the underestimate of non-harvest season survival led to an increase in the population estimate, and an underestimate of cub survival led to an increase in the population estimate while an overestimate led to decrease in the population estimate (Table 4).

Since the current model used by the WDNR is sensitive to the initial population estimate, we also performed two post hoc tests that assessed the sensitivity to extreme bias (50% increase and decrease) in the initial population estimate. The 50% underestimate had a PRC = −7.60% (CV = 7.67) for the population estimate, and the 50% overestimate had a PRC = 11.88% (CV = 12.03).

## Discussion

We fit age-at-harvest data from 2009 to 2016 to a Bayesian state-space model to create an accurate and precise estimate of black bear population abundance in Wisconsin. To assess the relative accuracy of our model we compared the 2011 abundance estimate to an independent capture-recapture population estimate from 2011^26^ (Fig. 4), and compared the abundance trend of our model to the trend of catch-per-unit-effort for the state. We found a strong correlation in trend with the catch-per-unit-effort from the state, with both estimating a decreasing trend, and similar abundance estimates to the independent abundance estimate from 2011. Our model for black bears appears to be a marked improvement on the population estimation model currently used by the WDNR (e.g.,^22^), by increasing the precision of the population estimate, providing estimates of variance in the estimate, and being independent of the initial population size values. By using Bayesian analyses, we allowed our model to use our prior information to create accurate posterior estimates, which can vary among years and age classes. Our population estimates were also generally robust to bias in the prior distributions for all parameters, except for reporting rate. These results support previous conclusions about the usefulness and applicability of Bayesian state-space models using age-at-harvest data for population estimation (e.g.,^11,13^), but now extend to situations lacking auxiliary demographic or other data from the population. Our state-space model appears to be a valid proof of concept for modeling wildlife populations; and Bayesian state-space models are a valuable tool to be added to the available analytical techniques for populations.

A strength of our state-space model was its robustness to biased prior distributions, including initial population size. The PRC values for all parameters were <2%, except for reporting rate (Table 4), and even a 50% biased estimate of initial population size led to PRC values of <12%. This is encouraging, because we derived many of our prior distributions from literature values and lack of information about parameter values can cause problems in many population estimation models (e.g.,^2^), especially when models are sensitive to parameters that are determined by expert opinion that can itself be biased^9^. We primarily used parameters that were derived from literature review from black bear studies in the northern mixed forest ecotone. These are informed values that help the model perform better than completely uninformed parameters and similar data are generally available for most harvested species across North America. Many population models, especially deterministic models, are sensitive to initial population size^16^, but being robust to bias in these estimates is a key strength of this model, especially when considered for use by management agencies. Considering how robust the state-space model is to biased prior distributions, and the applicability of using prior distributions informed by the literature review, the priority for future work should focus on accurately determining the reporting rates, potentially in the form of surveys.

Age-at-harvest models are clearly dependent on the quality of age-at-harvest data available to fit to the model. Our model was robust to bias in prior distributions partly because the quality of age-at-harvest data collected for bears in Wisconsin is excellent and broken down into specific age classes rather than general age stages (juvenile, yearling, adult). Consequently, survival probability was well-estimated in our model. Population models, especially for long-lived species such as black bears, often are most sensitive to adult, particularly female, survival probability (e.g.,^2,16^). Non-harvest mortality for black bears is typically low^38,39^, and therefore the focus of most research is on harvest survival. Our model inference supports this focus, in that estimates for non-harvest survival were considerably greater than for harvest-season survival, even though the harvest season is much shorter than the non-harvest season^22^. We did not account for the potential of additive versus compensatory mortality, but this should be considered in future analyses. Our state-space model, however, shows that harvest season survival can be precisely estimated using only age-at-harvest data, assuming quality data are available, and informative prior distributions on other parameters can be reasonably constructed. In cases where less age-at-harvest data is available, auxiliary data can be integrated into the model to potentially improve the precision of the estimates. Examples of data that can be incorporated include annual independent population estimates or observation surveys, survival estimates or other demographic parameters, or other covariates that affect demographic parameters such as winter severity or snow depth^13^. These results underscore the usefulness of sex and age data that are collected by many management agencies for harvested animals, and agencies interested in using state-space models to estimate populations should continue to collect this information.

The posterior distributions for recruitment variables were similar to the prior distributions, indicating that our prior beliefs were not updated by the model. The lone parameter that used data from Wisconsin (other than initial population size) was interbirth interval and proportion of age at first litter data (from^30^), therefore, the litter size values from the literature could potentially have underestimated litter sizes in Wisconsin. Black bear fecundity is strongly linked to food^17,19^, with heavier and older females producing more cubs^40–43^, particularly those with access to human foods^17^. There are few restrictions on the amount or frequency of bait that can be placed for black bears in Wisconsin^44^, which differs from some other jurisdictions, and as a result >40% of food consumed by the bear population is from intentional bait^44^. Access to this extra nutrition may lead to relatively larger litter sizes in Wisconsin compared to other areas, and therefore may lead to higher fecundity rates than currently reported in the literature. Given the robustness of the model to bias in fecundity parameters, however, this may not greatly affect the abundance estimates.

We based the initial proportions of bears in each sex and age class on the proportion of the harvest for each class. The prior distributions we used were reasonable, but were improved by the estimated posterior distribution. The posteriors were generally slightly lower than the prior distributions for males and generally slightly higher for females (Supplementary Material 5h). This is likely due to male bears being more frequently harvested^45^, and these proportions in the prior distributions can be adjusted to account for the potential bias we introduced, which would likely allow the model to converge more quickly. By using a Bayesian modeling framework, the model was able to account for potential bias in the prior values, which is important when assuming that the harvest among age classes is proportional to their abundance in the population. When implementing the model for management and conservation, instead of using the independent population estimates to proof the model abundance estimate, we suggest using the independent population estimates as the starting population values. It is also important to perform independent population estimates every 3–5 years to improve the model precision over time, and ensure the abundance estimates are realistic (e.g.,^22^).

Although our model is a valid proof of concept for updating population estimation in Wisconsin and other states, management agencies should adjust and fine-tune the model to match regionally and management zone specific conditions before using for management and setting quotas. This model is based on a statewide data, and produces only statewide estimates, but most states (including Wisconsin) are split into management zones. Management models should be split into an estimate for each zone, and managers can consider setting zone-specific prior distributions based on the unique ecology and hunting culture of each zone. The state-space model allows for precise estimates of wildlife populations, including for K-selected species which are vulnerable to over-harvest, making it valuable in both management and conservation settings. Due to budgetary constraints, many agencies are considering ways to reduce spending, but our model has shown the value of long-term age-at-harvest datasets.

Our harvest model was for black bears, but a similar model can be built for other harvested species, and, if needed, other data can be integrated into the model to increase the accuracy of the population estimate. Bayesian state-space models have now been successfully used for black bears and white-tailed deer (*Odocoileus virginianus*^13^), and similar models could be used for other harvested species that have a reasonable number of individuals in the harvest with known sex and age. Our model worked well partly because the WDNR has attempted to age and sex every harvested bear, but the state-space models also perform well when only a small proportion (e.g., 5%) of animals are aged^13^. Most management agencies have collected sex and age data for harvested animals over the course of decades, and our model should be widely applicable to agencies. In addition, we were able to create reasonable population estimates without using auxiliary data, which is a step forward for population models. Importantly, Bayesian state-space models are flexible, and can be adjusted to any harvest system, including those with unique data or parameters.

## Electronic supplementary material

Supplementary Material

Supplementary Material 2a

Supplementary Material 2b

Supplementary Material 3a

Supplementary Material 3b

Supplementary Material 3c

Supplementary Material 4

Supplementary Material 5

## References

[1] Leopold, A. *Game management*. (University of Wisconsin Press, 1986).

[2] Skalski, J. R., Ryding, K. E. & Millspaugh, J. J. *Wildlife demography: analysis of sex, age, and count data*. (Elsevier Academic Press, 2005).

[3] Fieberg, J. R., Shertzer, K. W., Conn, P. B., Noyce, K. V. & Garshelis, D. L. Integrated population modeling of black bears in minnesota: implications for monitoring and management. *PLoS One* **5** (2010). 10.1371/journal.pone.0012114 PMC2920827 20711344

[4] Gese, E. M. Monitoring of terrestrial carnivore populations. *Carniv. Conserv*. 372–396 (2001).

[5] Hiller TL, Etter DR, Belant JL, Tyre AJ (2011). Factors affecting harvests of fishers and American martens in northern michigan. J. Wildl. Manage..

[6] Karanth KU, Nichols JD (1998). Estimation of tiger densities in India using photographic captures and recaptures. Ecology.

[7] Rich LN (2013). Estimating occupancy and predicting numbers of gray wolf packs in Montana using hunter surveys. J. Wildl. Manage..

[8] Allen ML, Wittmer HU, Setiawan E, Jaffe S, Marshall AJ (2016). Scent marking in Sunda clouded leopards (*Neofelis diardi*): Novel observations close a key gap in understanding felid communication behaviours. Sci. Rep..

[9] Hristienko H, McDonald JEM (2007). Going into the 21st century: a perspective on trends and controversies in the management of the American black bear. Ursus.

[10] Caswell, H. *Matrix population models: construction, analysis, and interpretation*. (Sinauer Associates, 2001).

[11] Conn PB, Diefenbach DR, Laake JL, Ternent MA, White GC (2008). Bayesian analysis of wildlife age-at-harvest data. Biometrics.

[12] Skalski JR (2011). Abundance trends of American martens in Michigan based on statistical population reconstruction. J. Wildl. Manage..

[13] Norton, A. S. Integration of harvest and time-to-event data used to estimate demographic parameters for white-tailed deer. *Ph.D. Dissertation* (University of Wisconsin, Madison, 2015).

[14] White GC, Lubow BC (2002). Fitting population models to multiple sources of observed data. J. Wildl. Manage..

[15] Millspaugh JJ (2009). An evaluation of sex-age-kill (SAK) model performance. J. Wildl. Manage..

[16] Grund MD, Woolf A (2004). Development and evaluation of an accounting model for estimating deer population sizes. Ecol. Modell..

[17] Rogers LL (1987). Effects of food supply and kinship on social behavior, movements, and population growth of black bears in northeastern Minnesota. Wildl. Monogr..

[18] Taylor AP, Allen ML, Gunther MS (2015). Black bear marking behaviour at rub trees during the breeding season in northern California. Behaviour.

[19] Noyce KV, Garshelis DL (1994). Body size and blood characteristics as indicators of condition and reproductive performance in black bears. Bears Their Biol. Manag..

[20] Obbard ME, Howe EJ (2008). Demography of black bears in hunted and unhunted areas of the boreal forest of Ontario. J. Wildl. Manage..

[21] Garshelis, D. L., Scheick, B. K., Doan-Crider, D. L., Beecham, J. J. & Obbard, M. E. *Ursus americanus*. *IUCN Red List Threat. Species* **8235**, IUCN 2008: T41687A114251609 (2016).

[22] MacFarland, D. M. Population estimation, habitat associations and range expansion of black bears in the upper midwest. *Ph.D. Dissertation* (University of Wisconsin, 2009).

[23] Sadeghpour MH, Ginnett TF (2011). Habitat selection by female American black bears in northern Wisconsin. Ursus.

[24] MacFarland DM, Van Deelen TR (2011). Using simulation to explore the functional relationships of terrestrial carnivore population indices. Ecol. Modell..

[25] Garshelis DL, Hristienko H (2006). State and provincial estimates of American black bear numbers versus assessments of population trend. Ursus.

[26] Rolley, R. E. & Macfarland, D. M. *Black Bear Population Analyses 201*4 (2014).

[27] Beston JA (2011). Variation in life history and demography of the American black bear. J. Wildl. Manage..

[28] Bailey RG (1983). Delineation of ecosystem regions. Environ. Manage..

[29] Stoneberg RP, Jonkel CJ (1966). Age determination of black bears by cementum layers. J. Wildl. Manage..

[30] Allen ML, Kohn B, Roberts NM, Crimmins SM, Van Deelen TR (2017). Benefits and drawbacks of determining reproductive histories for black bears (*Ursus americanus*) from cementum annuli techniques. Can. J. Zool..

[31] Kolenosky GB (1986). The effects of hunting on an Ontario black bear population. Bears Their Biol. Manag..

[32] Buckland ST, Newman KB, Thomas L, Koesters NB (2004). State-space models for the dynamics of wild animal populations. Ecol. Modell..

[33] Newman KB, Fernández C, Thomas L, Buckland ST (2009). Monte Carlo inference for state-space models of wild animal populations. Biometrics.

[34] R Core Team. R: The R Project for Statistical Computing. Available at: https://www.r-project.org/. (Accessed: 28th March 2018) (2017).

[35] Plummer M (2003). JAGS: A Program for Analysis of Bayesian Graphical Models Using Gibbs Sampling. Proc. 3rd Int. Work. Distrib. Stat. Comput..

[36] Plummer, M. rjags: Bayesian graphical models using MCMC. R package version 2.2. 0–4 (2011).

[37] Gelman A, Rubin DB (1992). Inference from iterative simulation using multiple sequences. Stat. Sci..

[38] Beringer J (1998). The influence of a small sanctuary on survival rates of black bears in North Carolina. J. Wildl. Manage..

[39] Lee DJ, Vaughan MR (2005). Yearling and subadult black bear survival in a hunted Virginia population. J. Wildl. Manage..

[40] Eiler JH, Wathen WG, Pelton MR (1989). Reproduction in black bears in the southern Appalachian Mountains. J. Wildl. Manage..

[41] Kolenosky GB (1990). Reproductive Biology of Black Bears in East-Central Ontario. Bears Their Biol. Manag..

[42] Samson C, Huot J (1995). Reproductive biology of female black bears in relation to body mass in early winter. J. Mammal..

[43] Stringham SF (1990). Black bear reproductive rate relative to body weight in hunted populations. Bears Their Biol. Manag..

[44] Kirby R, Macfarland DM, Pauli JN (2017). Consumption of intentional food subsidies by a hunted carnivore. J. Wildl. Manage..

[45] Malcolm KD, Van Deelen TR (2010). Effects of habitat and hunting framework on American black bear harvest structure in Wisconsin. Ursus.

[46] Bridges AS, Vaughan MR, Fox JA (2011). American black bear estrus and parturition in the Alleghany Mountains of Virginia. Ursus.

[47] McLaughlin CR, Matula GJJ, O’Connor RJ (1994). Synchronous reproduction by Maine black bears. Bears Their Biol. Manag..

[48] Mcdonald JE, Fuller TK (2001). Prediction of litter size in American black bears. Ursus.

[49] Elowe KD, Dodge WE (1989). Factors affecting black bear reproductive success and cub survival. J. Wildl. Manage..

[50] Kasbohm JW, Vaughan MR, Kraus JG (1996). Effects of gypsy moth infestation on black bear reproduction and survival. J. Wildl. Manage..

[51] Hellgren EC, Vaughan MR (1989). Demographic analysis of a black Bear population in the Great Dismal Swamp. J. Wildl. Manage..

[52] Diefenbach DR, Laake JL, Alt GL (2004). Spatio-temporal and demographic variation in the harvest of black bears: implications for population estimation. J. Wildl. Manage..

[53] Noyce KV, Garshelis DL (1997). Influence of natural food abundance on black bear harvests in Minnesota. J. Wildl. Manage..

[54] Powell RA, Zimmerman JW, Seaman DE, Gilliam JF (1996). Demographic analyses of a hunted black bear population with access to a refuge. Conserv. Biol..

[55] Brongo LL, Mitchell MS, Grand JB (2005). Long-term analysis of survival, fertility, and population growth rate of black bears in North Carolina. J. Mammal..

