# Supplementary material for: A Bayesian state-space model using age-at-harvest data for estimating the population of black bears (Ursus americanus) in Wisconsin
Source: Sci Rep. 2018 Aug 20;8:12440. doi: 10.1038/s41598-018-30988-4 (PMC6102245; doi:10.1038/s41598-018-30988-4)
Supplement: Supplementary file 2 — Supplementary Material 2a [file 41598_2018_30988_MOESM2_ESM.pdf]

A Bayesian state-space model using age-at-harvest data for estimating the population of black bears (*Ursus americanus*) in Wisconsin

Maximilian L. Allen, Andrew S. Norton, Glenn Stauffer, Nathan M. Roberts, Yanshi Luo, Qing Li, David MacFarland, and Timothy R. Van Deelen

Supplementary Information 2a. The results of the 2009 Wisconsin Department of Natural Resources population analysis.

Black Bear Population Analyses
2009

By Robert E. Rolley and Michael L. Worland

Abstract

Bear visitation rates averaged 58% for 18 bait station surveys conducted in the primary range (Zones A, B, and D), and 31% for 7 surveys conducted in the peripheral range (Zone C). Revised population models produced a statewide estimate of approximately 21,500 bears in Fall, 2009. A harvest of 4,585 bears was approved by the Natural Resources Board for the 2009 season.

Methods

Bear bait station surveys were conducted by wildlife management and research personnel in the 18 counties comprising the primary bear range and 7 counties within the peripheral range in 2009. The surveys were run between 15 June and 15 July, and consisted of 50 bait stations placed at 0.5-mile intervals along drivable roads. A plastic mesh overwrap bag filled with approximately 2 lb. of fresh meat was securely wired to a tree about 7 ft above the ground at each bait station. Bait stations were checked for bear visitations after 7 nights.

A station was considered to have been visited by bears if the bag of meat was gone and the wire securing it had been stretched or broken, or by marks on the trees and/or trails leading to the station. Bait stations were considered inoperable and not included in the calculations if they could not be found or if animals other than bears had taken the bait.

Three-year running average visitation rates ([year x 2 + $year^{+1}$]/3 for first year; [$year^{-1}$ + year x 2]/3 for last year, and [$year^{-1}$ + year + $year^{+1}$]/3 for all other years) were used as an index to bear population trends. Combining years reduced annual fluctuations resulting from small sample sizes and annual changes in the abundance of natural foods.

All harvested bears are required to be registered at DNR or cooperative stations. An upper first premolar was collected as the bears were registered, and the sex and county of kill were recorded for each bear. Registration personnel were provided instructions and envelopes for storing the teeth. Teeth were sent to the Matson's Lab in Milltown, MT for processing, and ages were assigned by counting annuli in the cementum.

Wisconsin's Bear Population Model was adapted from one developed and used in Minnesota (Garshelis 1990). The model was updated in 2008 to include the most recent bear harvest, age, and bait station data, and used to estimate bear populations in each Bear Management Zone (Figure 1). Starting population size in the model was increased in all zones in 2008 to improve the correlation between model simulated population trends and trends in bait-station visitations and in consideration of the results of the tetracycline mark-recapture study conducted by MacFarland (2009).

Results

Bear visitation rates in the 2009 bait station survey averaged 73% in Zone A, 53% in Zone B, 44% in Zone D, and 58% in the primary bear range (Zones A, B, and D combined) (Table 1). Bear visitation rates in Zone C (peripheral range) averaged 31%.

The 3-year mean visitation rates in the primary bear range increased steadily during the mid 1980s and early 1990s, was fairly stable during the mid-to-late 1990s, and then slowly increased during the 2000s (Fig. 2). In the peripheral range (Zone C), bait station data suggests a substantial increase in the bear population during the late 1990s and early 2000s; 3-year average visitation rates doubled from 17% to 35% during 1996-2004. Visitation rates have since stabilized or declined in recent years in Zone C.

Teeth were collected from 2,691 of the 2,955 bears harvested in 2008. Age data from bears harvested in 2008 are not available at this time. The age structure of female bears harvested during 1986-2007 has been relatively stable (Table 2); mean age of harvested female bears averaged 4.6 years (range 4.2 - 5.3). The age structure of harvested male bears has tended to shift to a slightly younger distribution over the last 20 years with the mean age of harvested males declining from about 4 years to less than 3.5 years over the period.

Adjustments made in 2008 to the starting population size for the bear population model in all zones improved the correlation between simulated population trends and trends in bait-station visitations (Fig. 2). The models produced a statewide population estimate of approximately 21,450 bears in Fall, 2009 (Table 3). This included 9,300 bears in Zone A, 4,500 in Zone B, 2,750 in Zone C, and 4,900 in Zone D., The 2009 population estimates equate to bear densities of 1.6 bears/mi^2 of bear range in Zone A, 0.8 bears/mi^2 in Zone B, 0.3 bears/mi^2 of occupied range in Zone C, and 0.9 bears/mi^2 in Zone D.

Discussion

MacFarland (2009) estimated the statewide bear population of bears in autumn 2006 to be 33,657 $\pm$ 7,042 bears >1 year old based on a tetracycline mark-recapture study. This was considerably higher than the prior model-based estimates for 2006 (11,100 yearlings and adults, 14,300 bears including cubs) and was also higher than the revised model-based estimates for 2006 (15,450 yearlings and adults, 19,800 bears including cubs). Reasons for this difference are unclear. MacFarland (2009) suggested that the tetracycline mark-recapture estimate could be biased high due to violation of the assumption of equal capture probability. Alternatively he suggested that model-based estimates that are calibrated to bait-station indices could be biased low if the functional relationship between population size and the index is non-linear (i.e., the rate of increase of the population is faster than that indicated by the bait-station index). Limited auxiliary data (harvest success rates, bear damage complaints, bear observations by DNR staff) suggest that the bear population has been stable to slowly increasing since the early 1990s, consistent with the population trend suggested by the bait-station index. Additional research will be needed to resolve the difference between the mark-recapture and model-based population estimates.

The Natural Resources Board approved a harvest quota of 4,585 bears for the 2009 season. This included 1,700 bears in Zone A, 1,075 in Zone B, 860 in Zone C, and 950 in Zone D.

Literature Cited

Garshelis, D. L. 1990. Monitoring effects of harvest on black bear populations in North America: a review and evaluation of techniques. East Workshop Black Bear Res. and Manage. 10:120-144.

MacFarland, D. M. 2009. Population estimation, habitat associations and range expansion of black bears in the upper Midwest. Ph.D. dissertation. University of Wisconsin, Madison.

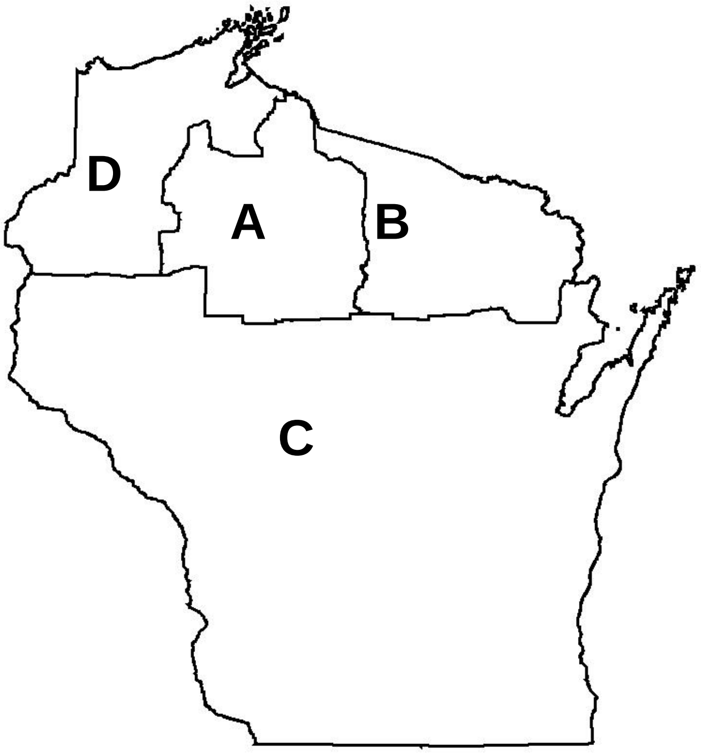

Figure 1. *Wisconsin's Black Bear Management Zones, 2009.*

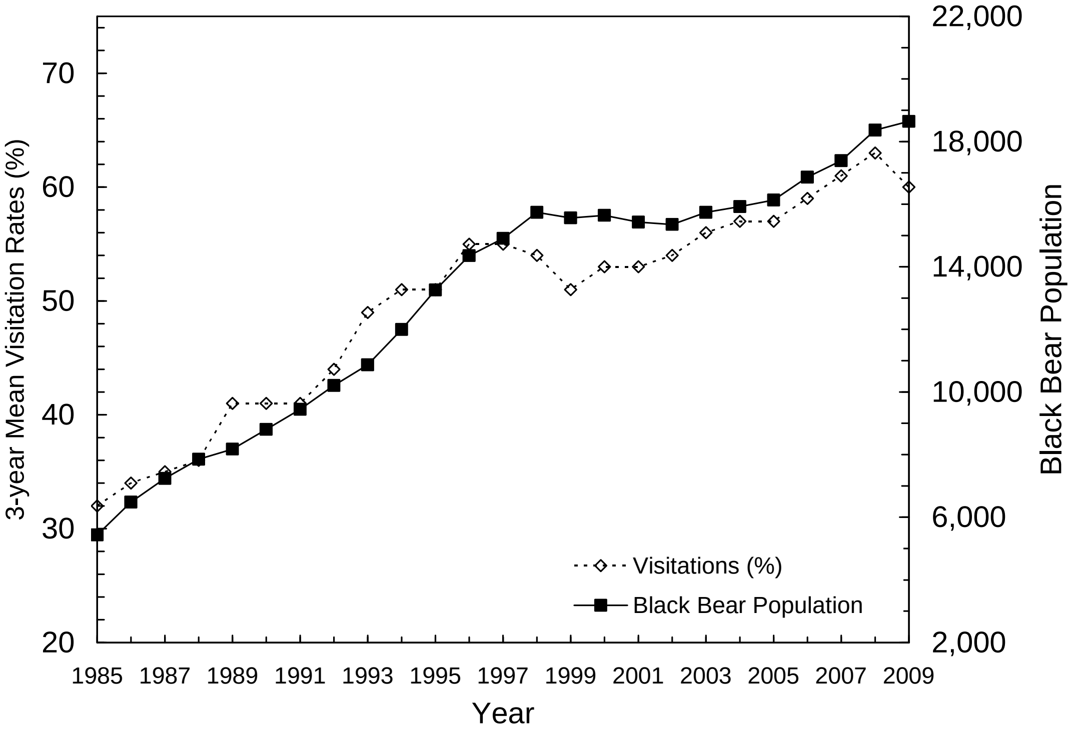

Figure 2. *Bear visitation rates on bait station surveys (3-yr running average) and population estimates calculated by the revised models for the primary range (Zones A, B, and D), 1985-2009.*

Table 1. *Percent of bear bait stations visited by bears, 1998-2009.*

County	1998	1999	2000	2001	2002	2003	2004	2005	2006	2007	2008	2009
Ashland	48	68	82	63	51	57	86	71	76	52	59	66
Bayfield	83	83	67	64	79	65	46	75	52	76	67	44
Burnett	63	60	71	84	53	36	32	46	43	37	35	5
Douglas	37	62	61	30	33	33	30	30	30	41	73	49
Florence	46	64	54	----	34	53	67	83	66	86	75	68
Forest	26	43	61	55	58	60	74	62	63	88	78	76
Iron	58	48	41	42	47	55	79	64	69	71	58	88
Langlade	29	30	48	44	56	53	54	63	53	44	46	48
Lincoln	52	41	55	33	68	44	27	30	39	73	61	64
Marinette	26	44	35	39	65	24	47	50	48	64	31	32
Oconto	6	18	6	25	47	28	31	23	17	23	53	39
Oneida	23	66	23	36	63	95	70	48	54	57	54	39
Price	43	31	50	50	42	68	78	26	33	50	66	69
Rusk	71	84	84	91	72	58	80	98	68	98	92	92
Sawyer	66	76	68	91	91	79	67	90	77	87	80	70
Taylor	62	52	42	36	50	57	58	90	66	92	86	89
Vilas	36	52	31	34	26	47	33	32	56	39	68	34
Washburn	60	90	91	74	88	85	84	92	70	88	87	70
**Primary Range**	**47**	**56**	**51**	**52**	**56**	**54**	**58**	**60**	**53**	**65**	**65**	**58**
Barron	26	11	30	28	17	11	20	30	5	3	19	40
Chippewa	15	52	41	20	44	50	42	47	17	35	44	40
Clark	12	33	16	39	54	52	64	48	28	45	47	55
Jackson	27	0	28	11	20	15	14	8	24	13	14	13
Marathon	7	8	13	32	66	69	65	53	45	38	51	42
Menominee	8	5	46	6	11	9	35	14	0	6	26	9
Polk	8	4	9	7	2	---	---	---	---	---	---	---
Shawano	0	0	0	7	0	---	11	3	0	0	4	10
**Peripheral Range**	**13**	**14**	**23**	**19**	**27**	**37**	**38**	**30**	**18**	**22**	**31**	**31**

Table 2. *Age classes of bears harvested in Wisconsin, 1986-2006*
.

Year	Sex	Percent in age class			No. aged	Mean age
		1-2 yr	3-9 yr	10+ yr		
1986	Male	59.5	37.2	3.3	210	3.6
	Female	43.8	41.3	9.9	121	4.2
1987	Male	52.6	43.2	4.2	401	4.1
	Female	41.5	52.0	6.5	200	4.6
1988	Male	60.4	35.0	4.6	439	3.7
	Female	40.9	51.9	7.2	345	4.7
1989	Male	53.9	39.0	7.1	397	4.2
	Female	42.5	47.9	9.6	261	5.0
1990	Male	67.0	30.4	2.6	454	3.4
	Female	46.8	48.1	5.1	331	4.6
1991	Male	56.9	37.3	5.8	448	4.0
	Female	38.9	54.9	6.2	306	4.7
1992	Male	63.9	32.1	4.0	474	3.5
	Female	48.4	45.0	6.6	380	4.3
1993	Male	50.9	41.7	7.4	405	4.3
	Female	37.8	57.3	4.9	286	4.6
1994	Male	62.6	31.4	6.0	441	3.9
	Female	50.9	45.0	4.1	271	4.2
1995	Male	55.7	41.4	2.9	600	3.6
	Female	37.7	52.0	10.5	435	5.3
1996	Male	60.0	37.3	2.7	771	3.6
	Female	46.8	45.6	7.6	536	4.7
1997	Male	65.0	32.6	2.5	765	3.5
	Female	47.9	44.2	7.9	620	4.6
1998	Male	65.0	33.4	1.6	1,134	3.3
	Female	49.0	44.2	6.9	904	4.5
1999	Male	67.6	29.9	2.4	1,058	3.3
	Female	51.5	39.3	9.2	954	4.7
2000	Male	68.1	29.0	2.9	1,227	3.3
	Female	49.8	41.5	8.7	1,046	4.7
2001	Male	67.8	29.2	3.0	1,250	3.4
	Female	51.2	40.8	8.0	1,023	4.6
2002	Male	59.5	34.6	5.9	1,094	3.9
	Female	44.5	43.7	11.8	946	5.2
2003	Male	64.3	33.3	2.4	1,349	3.1
	Female	48.4	43.0	8.2	1,065	4.6
2004	Male	62.9	33.9	7.9	1,332	3.2
	Female	48.4	43.7	3.2	1,177	4.3
2005	Male	57.1	40.1	2.8	1,267	3.4
	Female	44.7	47.8	7.6	898	4.5
2006	Male	58.8	38.7	2.5	1,421	3.4
	Female	44.8	47.0	8.2	1,258	4.6

Table 3. *Modeled bear population estimates by Management Zone, 1988-2009[a].*

Year	Bear Management Zone				State
	A	B	C	D[b]	
1988	3,600	1,600	700	2,700	8,600
1989	3,600	1,700	750	2,900	8,950
1990	3,800	1,900	900	3,100	9,700
1991	4,000	2,000	950	3,500	10,450
1992	4,300	2,100	1,020	3,800	11,250
1993	4,400	2,200	1,100	4,200	11,900
1994	4,800	2,400	1,200	4,800	13,200
1995	5,400	2,600	1,350	5,300	14,650
1996	6,100	2,700	1,450	5,600	15,850
1997	6,400	2,800	1,550	5,700	16,450
1998	6,900	2,900	1,700	5,900	17,400
1999	6,800	3,000	1,800	5,700	17,300
2000	6,900	3,200	1,950	5,600	17,650
2001	6,800	3,300	2,150	5,300	17,550
2002	6,700	3,500	2,300	5,200	17,700
2003	7,000	3,700	2,600	5,000	18,300
2004	7,200	4,000	2,750	4,800	18,750
2005	7,600	4,000	2,800	4,600	19,000
2006	8,100	4,200	2,900	4,600	19,800
2007	8,700	4,200	2,850	4,500	20,250
2008	9,300	4,400	2,850	4,600	21,150
2009	9,300	4,500	2,750	4,900	21,450

[a] Population estimates for all zones and the statewide estimates differ from those previously reported due to adjustments to the starting population size in the zone models.

[b] Formally Zone A1.
