# Supplementary material for: A Bayesian state-space model using age-at-harvest data for estimating the population of black bears (Ursus americanus) in Wisconsin
Source: Sci Rep. 2018 Aug 20;8:12440. doi: 10.1038/s41598-018-30988-4 (PMC6102245; doi:10.1038/s41598-018-30988-4)
Supplement: Supplementary file 3 — Supplementary Material 2b [file 41598_2018_30988_MOESM3_ESM.pdf]

A Bayesian state-space model using age-at-harvest data for estimating the population of black bears (*Ursus americanus*) in Wisconsin

Maximilian L. Allen, Andrew S. Norton, Glenn Stauffer, Nathan M. Roberts, Yanshi Luo, Qing Li, David MacFarland, and Timothy R. Van Deelen

Supplementary Information 2b. The results of the 2017 Wisconsin Department of Natural Resources population analysis.

Black Bear Population Analyses
2017

By Catherine C. Dennison, Nathan M. Roberts and David M. MacFarland

Abstract

Bear visitation rates averaged 55% for 17 bait station surveys conducted in the primary range (Zones A, B, and D), and 25% for 7 surveys conducted in the peripheral range (Zone C). Revised population models produced a statewide estimate of approximately 28,600 bears in fall 2017. A harvest of 5,000 bears was approved by the Natural Resources Board for the 2017 season.

Methods

Bear bait station surveys were conducted by wildlife management and research personnel in the 17 counties comprising the primary bear range and 7 counties within the peripheral range in 2017. The surveys were run between 15 June and 15 July, and consisted of 50 bait stations placed at 0.5-mile intervals along drivable roads. A plastic mesh overwrap bag filled with approximately 2 lb. of fresh meat was securely wired to a tree about 7 feet above the ground at each bait station. Bait stations were checked for bear visitations after 7 nights.

A station was considered to have been visited by bears if the bag of meat was gone and the wire securing it had been stretched or broken or by marks on the trees and/or trails leading to the station. Bait stations were considered inoperable and not included in the calculations if they could not be found or if animals other than bears had taken the bait.

Three-year running average visitation rates ([year x 2 + $year^{+1}$]/3 for first year; [$year^{-1}$ + year x 2]/3 for last year, and [$year^{-1}$ + year + $year^{+1}$]/3 for all other years) were used as an index to bear population trends. Combining years reduced annual fluctuations resulting from small sample sizes and annual changes in the abundance of natural foods.

Registration is required for all harvested bears. Registration is completed electronically by phone, computer or at a cooperating station. An upper first premolar, submitted by mail, is required to complete legal registration. The sex and county of kill were recorded for each bear. Teeth were sent to the Matson’s Lab in Milltown, MT for processing, and ages were assigned by counting annuli in the cementum.

Wisconsin’s Bear Population Model was adapted from one developed and used in Minnesota (Garshelis 1990). Zone-specific models were updated in 2014 to include the most recent bear harvest, age, and bait station data, and were used to estimate bear population size in each Bear Management Zone (Figure 1). Starting population size in the models was adjusted in all zones in 2013 based on estimates from the tetracycline mark-recapture study conducted in 2011 and 2012 (MacFarland 2009, Rolley et al. 2014). Previously models were calibrated to maximize the correlation between model simulated population trends and trends in bait-station visitations.

Results

Bear visitation rates in the 2017 bait station survey averaged 67% in Zone A, 42% in Zone B, 52% in Zone D, and 55% in the primary bear range (zones A, B, and D combined) (Table 1). Bear visitation rates in Zone C (peripheral range) averaged 25%.

The 3-year mean visitation rates in the primary bear range increased steadily during the mid-1980s and early 1990s, was fairly stable during the mid-to-late 1990s, slowly increased during the 2000s, declined during the late 2000s, and has been relatively stable since 2012(Fig. 2). In the peripheral range (Zone C), bait station data suggests a substantial increase in the bear population during the late 1990s and early 2000s; 3-year average visitation rates doubled from 17% to 35% during 1996-2004. Visitation rates during the last 10 years in Zone C were relatively stable.

Teeth were collected from 3,852 of the 4,682 bears harvested in 2016. The age structure of female bears harvested during 1993-2016 has been relatively stable (Table 2); mean age of harvested female bears averaged 4.6 years (range 3.7 - 5.3). The age structure of harvested male bears has shifted to a younger distribution over the last 24 years with the mean age of harvested males declining from approximately 4 years to about 3 years over the period.

Zone-specific models were calibrated to yield estimates of the 2011 fall population that closely matched estimates from the 2011 tetracycline mark-recapture study. The models produced a statewide population estimate of approximately 28,600 bears in fall 2017 (Table 3). This included 5,100 bears in Zone A, 5,200 in Zone B, 9.700 in Zone C, and 8,700 in Zone D. The 2017 population estimates equate to bear densities of 0.9 bears/mi^2 of bear range in Zone A, 1.0 bears/mi^2 in Zone B, 1.1 bears/mi^2 of occupied range in Zone C, and 1.6 bears/mi^2 in Zone D.

Discussion

Population models that were calibrated to the 2011 zone-specific mark-recapture estimates, together with trends in bait-station visitations, suggest that higher harvests since 2009 reduced the bear population in zone A and stabilized population growth in Zone B (Table 3). Our model for Zone D suggests that higher recent harvests in Zone D may be slowing population growth. The population model for Zone C suggests the population has continued to increase in recent years.

The Natural Resources Board approved a harvest quota of 5,000 bears for the 2017 season. This included 1,200 bears in Zone A, 900 in Zone B, 1,300 in Zone C, and 1,600 in Zone D.

Literature Cited

Garshelis, D.L. 1990. Monitoring effects of harvest on black bear populations in North America: a review and evaluation of techniques. East Workshop Black Bear Res. and Manage. 10:120-144.

MacFarland, D.M. 2009. Population estimation, habitat associations and range expansion of black bears in the upper Midwest. Ph.D. dissertation. University of Wisconsin, Madison.

Rolley, R.E., D.M. MacFarland, N.M. Roberts, and T.R. Pearson. 2014. Black Bear Population Analyses, 2014. Pages 28-34 in J. Kitchell and B. Dhuey (editors). Wisconsin Wildlife Surveys: August 2014. Wisconsin Department of Natural Resources, Bureau of Science Services, Madison, WI.

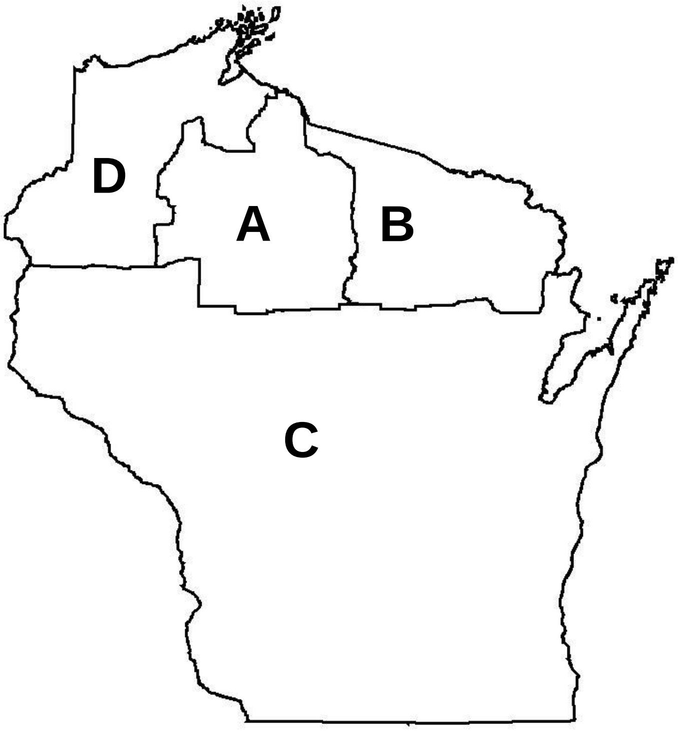

Figure 1. *Wisconsin's Black Bear Management Zones, 2017.*

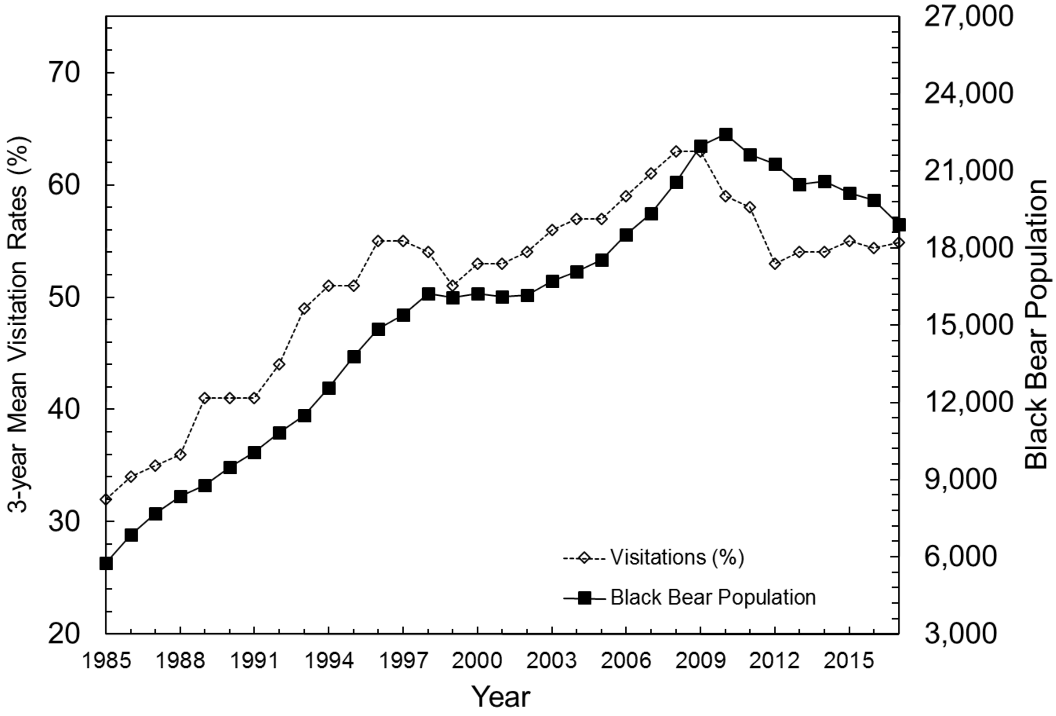

Figure 2. *Bear visitation rates on bait station surveys (3-yr running average) and pre-hunt population estimates calculated by the revised models for the primary range (Zones A, B, and D), 1985-2017.*

Table 1. *Percent of bear bait stations visited by bears, 2004-2017.*

County	2005	2006	2007	2008	2009	2010	2011	2012	2013	2014	2015	2016	2017
Ashland	71	76	52	59	66	33	42	27	32	69	65	81	41
Bayfield	75	52	76	67	44	67	64	56	53	69	39	40	56
Burnett	46	43	37	35	5	51	48	43	21	38	23	30	23
Douglas	30	30	41	73	49	63	33	50	38	48	43	48	N/A
Florence	83	66	86	75	68	79	30	59	79	79	56	56	43
Forest	62	63	88	78	76	84	58	76	57	82	55	47	65
Iron	64	69	71	58	88	77	94	76	30	23	63	58	56
Langlade	63	53	44	46	48	46	61	57	56	37	41	38	56
Lincoln	30	39	73	61	64	68	37	42	60	28	38	37	47
Marinette	50	48	64	31	32	57	34	41	23	27	31	34	24
Oconto	23	17	23	53	39	48	43	24	17	41	17	29	26
Oneida	48	54	57	54	39	62	49	60	55	61	70	74	76
Price	26	33	50	66	69	84	67	53	57	67	66	65	59
Rusk	98	68	98	92	92	93	78	98	88	79	72	94	90
Sawyer	90	77	87	80	70	60	60	46	51	64	59	51	61
Taylor	90	66	92	86	89	76	75	62	65	79	66	89	87
Vilas	32	56	39	68	34	41	28	29	40	26	62	41	20
Washburn	92	70	88	87	70	80	78	76	63	79	84	97	78
**Primary Range**	**60**	**53**	**65**	**65**	**58**	**66**	**54**	**55**	**51**	**56**	**53**	**55**	**55**
Barron	30	5	3	19	40	29	44	34	45	35	22	40	45
Chippewa	47	17	35	44	40	53	72	78	42	63	62	50	47
Clark	48	28	45	47	55	33	42	36	44	36	43	49	15
Jackson	8	24	13	14	13	15	20	11	3	10	8	22	0
Marathon	53	45	38	51	42	44	44	42	30	29	31	34	23
Menominee	14	0	6	26	9	13	---	---	---	38	16	18	39
Shawano	3	0	0	4	10	0	0	3	10	9	0	7	5
**Peripheral Range**	**30**	**18**	**22**	**31**	**31**	**27**	**32**	**34**	**30**	**32**	**28**	**32**	**25**

Table 2. *Age classes of bears harvested in Wisconsin, 1993-2016.*

Year	Sex	Percent in age class			No. aged	Mean age
		1-2 yr	3-9 yr	10+ yr		
1993	Male	50.9	41.7	7.4	405	4.3
	Female	37.8	41.7	7.4	405	4.3
1994	Male	62.6	31.4	6.0	441	3.9
	Female	50.9	45.0	4.1	271	4.2
1995	Male	55.7	41.4	2.9	600	3.6
	Female	37.7	52.0	10.5	435	5.3
1996	Male	60.0	37.3	2.7	771	3.6
	Female	46.8	45.6	7.6	536	4.7
1997	Male	65.0	32.6	2.5	765	3.5
	Female	47.9	44.2	7.9	620	4.6
1998	Male	65.0	33.4	1.6	1,134	3.3
	Female	49.0	44.2	6.9	904	4.5
1999	Male	67.6	29.9	2.4	1,058	3.3
	Female	51.5	39.3	9.2	954	4.7
2000	Male	68.1	29.0	2.9	1,227	3.3
	Female	49.8	41.5	8.7	1,046	4.7
2001	Male	67.8	29.2	3.0	1,250	3.4
	Female	51.2	40.8	8.0	1,023	4.6
2002	Male	59.5	34.6	5.9	1,094	3.9
	Female	44.5	43.7	11.8	946	5.2
2003	Male	64.3	33.3	2.4	1,349	3.1
	Female	48.4	43.0	8.2	1,065	4.6
2004	Male	62.9	33.9	7.9	1,332	3.2
	Female	48.4	43.7	3.2	1,177	4.3
2005	Male	57.1	40.1	2.8	1,267	3.4
	Female	44.7	47.8	7.6	898	4.5
2006	Male	58.8	38.7	2.5	1,421	3.4
	Female	44.8	47.0	8.2	1,258	4.6
2007	Male	61.0	36.6	2.3	1,367	3.3
	Female	42.0	48.3	9.7	1,135	4.8
2008	Male	58.1	38.5	3.4	1,456	3.6
	Female	42.9	49.0	8.0	1,169	4.7
2009	Male	59.6	38.4	2.0	1,794	3.3
	Female	45.6	47.2	7.3	1,523	4.4
2010	Male	68.5	30.0	1.4	2,144	2.9
	Female	50.0	42.1	7.9	2,190	4.3
2011	Male	61.4	34.6	4.0	1,882	3.4
	Female	42.2	47.1	10.8	1,786	5.0
2012	Male	70.7	27.3	2.1	2,984	3.0
	Female	49.0	41.5	9.6	2,171	4.5
2013	Male	60.0	36.4	3.6	1,884	3.4
	Female	42.5	46.2	11.3	1,753	4.9
2014	Male	69.2	28.9	1.9	2,098	2.9
	Female	50.9	41.6	7.6	2,047	4.3
2015	Male	66.5	31.6	2.0	1,777	3.0
	Female	45.8	44.3	9.9	1,650	4.5
2016	Male	66.9	30.9	2.2	1,839	2.4
	Female	50.1	41.9	8.0	1,945	3.7

Table 3. *Modeled bear population estimates by management zone, 1988-2017. Estimates are for fall, pre-hunt populations and include adults, yearlings, and cubs.*

Year	Bear Management Zone				State
	A	B	C	D	
1988	3,700	1,700	850	3,000	9,250
1989	3,700	1,900	950	3,200	9,750
1990	3,900	2,000	1,100	3,500	10,500
1991	4,100	2,100	1,150	3,800	11,150
1992	4,300	2,300	1,300	4,200	12,100
1993	4,400	2,400	1,400	4,600	12,800
1994	4,800	2,600	1,500	5,200	14,100
1995	5,300	2,800	1,650	5,700	15,450
1996	6,000	2,900	1,800	6,000	16,700
1997	6,300	3,000	1,950	6,100	17,350
1998	6,700	3,200	2,150	6,300	18,350
1999	6,600	3,300	2,350	6,200	18,450
2000	6,600	3,600	2,600	6,100	18,900
2001	6,500	3,800	2,900	5,800	19,000
2002	6,400	4,000	3,200	5,800	19,400
2003	6,600	4,300	3,650	5,800	20,350
2004	6,700	4,600	4,000	5,700	21,000
2005	7,000	4,700	4,300	5,800	21,800
2006	7,400	5,000	4,650	6,100	23,150
2007	7,800	5,200	4,850	6,300	24,150
2008	8,200	5,600	5,250	6,800	25,850
2009	8,600	5,800	5,650	7,500	27,550
2010	8,600	5,800	6,050	8,000	28,450
2011	7,700	5,700	6,400	8,200	28,000
2012	7,300	5,400	6,950	8,600	28,250
2013	6,300	5,200	7,500	8,900	27,900
2014	6,000	5,500	7,850	9,200	28,550
2015	5,600	5,500	8,350	9,100	28,550
2016	5,400	5,400	9,000	9,100	28,900
2017	5,100	5,200	9,700	8,700	28,700
