# Supplementary material for: A Bayesian state-space model using age-at-harvest data for estimating the population of black bears (Ursus americanus) in Wisconsin
Source: Sci Rep. 2018 Aug 20;8:12440. doi: 10.1038/s41598-018-30988-4 (PMC6102245; doi:10.1038/s41598-018-30988-4)
Supplement: Supplementary file 7 — Supplementary Material 4 [file 41598_2018_30988_MOESM7_ESM.pdf]

A Bayesian state-space model using age-at-harvest data for estimating the population of black bears (*Ursus americanus*) in Wisconsin

Maximilian L. Allen, Andrew S. Norton, Glenn Stauffer, Nathan M. Roberts, Yanshi Luo, Qing Li, David MacFarland, and Timothy R. Van Deelen

Supplementary Information 4. The observed harvest (***O***) and aged individuals (***C***) used in the model.

O

	Male	Female
2009	2158	1851
2010	2525	2608
2011	2191	2066
2012	2329	2317
2013	2035	1917
2014	2286	2240
2015	2198	2000
2016	2311	2371

C

A1

	1.5	2.5	3.5	4.5	5.5	6.5	7.5	8.5	9.5	10.5+
2009	370	316	261	127	107	72	74	36	36	108
2010	532	549	304	209	129	101	85	42	41	171
2011	401	349	226	155	139	96	95	54	69	189
2012	491	555	287	184	134	88	83	62	53	202
2013	406	334	281	150	105	73	86	56	55	198
2014	550	535	256	196	120	100	68	67	46	110
2015	391	364	262	155	101	90	54	40	29	163
2016	542	430	276	177	130	96	54	40	41	155

A2

	1.5	2.5	3.5	4.5	5.5	6.5	7.5	8.5	9.5	10.5+
2009	616	434	300	146	82	57	38	20	18	37
2010	823	633	270	197	63	47	25	14	17	31
2011	683	464	264	115	102	52	60	33	18	76
2012	784	710	248	142	69	51	31	27	15	44
2013	689	430	285	132	94	67	50	36	20	66
2014	859	604	274	144	77	51	21	19	24	32
2015	685	495	257	144	66	34	24	18	17	35

2016	755	471	265	134	74	48	23	10	10	41
