# Supplementary material for: A Bayesian state-space model using age-at-harvest data for estimating the population of black bears (Ursus americanus) in Wisconsin
Source: Sci Rep. 2018 Aug 20;8:12440. doi: 10.1038/s41598-018-30988-4 (PMC6102245; doi:10.1038/s41598-018-30988-4)

A Bayesian state-space model using age-at-harvest data for estimating the population of black bears (*Ursus americanus*) in Wisconsin

Maximilian L. Allen, Andrew S. Norton, Glenn Stauffer, Nathan M. Roberts, Yanshi Luo, Qing Li, David MacFarland, and Timothy R. Van Deelen

Supplementary Information 1. The four management zones used by WDNR for black bears in Wisconsin. The figure was created with ArcGIS 10.3 (www.arcgis.com) with a base map from the WDNR GIS open data portal (https://data-wi-dnr.opendata.arcgis.com/).

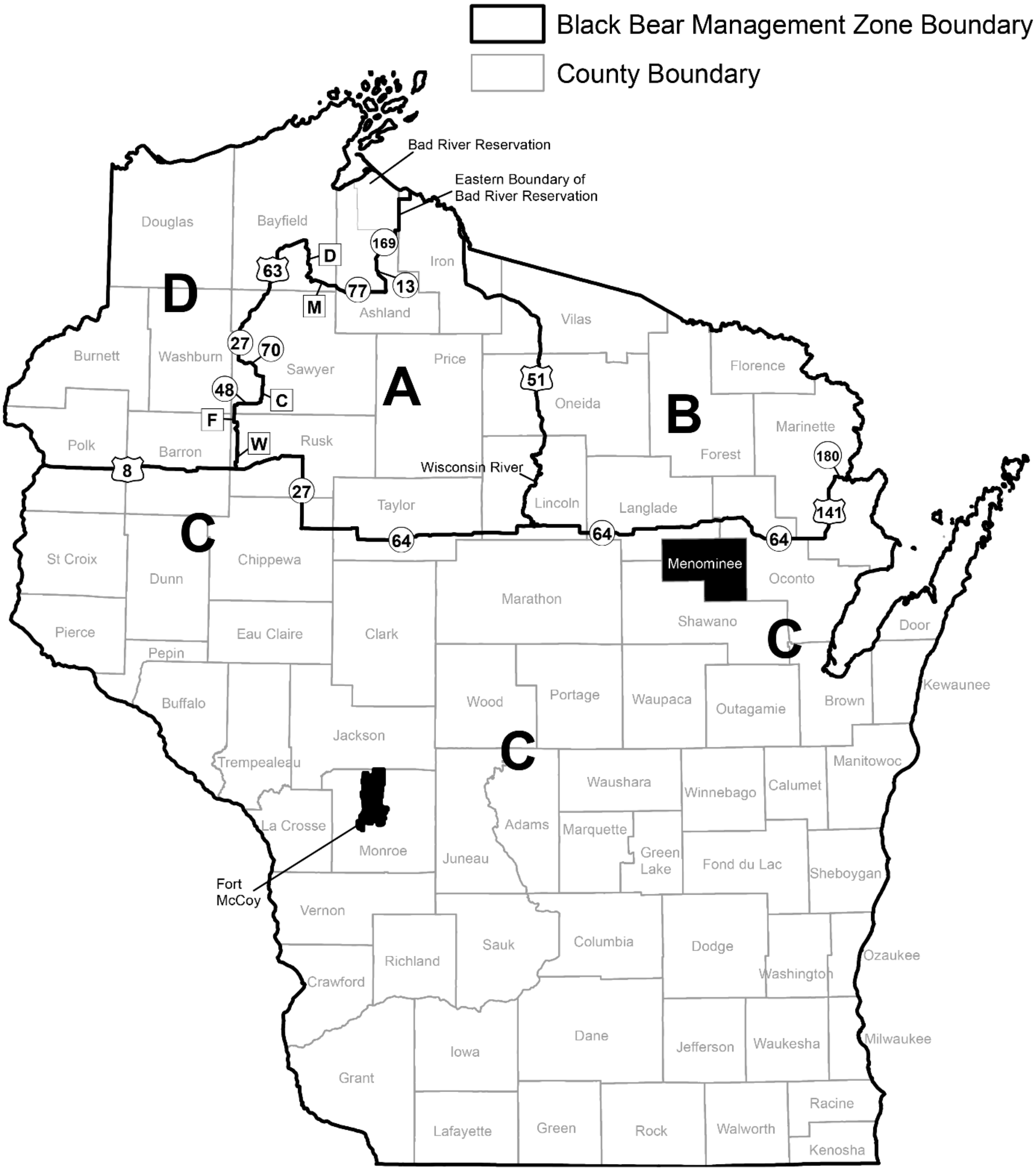

Supplement: Supplementary file 1 — Supplementary Material [file 41598_2018_30988_MOESM1_ESM.pdf]
