# Supplementary material for: A Bayesian state-space model using age-at-harvest data for estimating the population of black bears (Ursus americanus) in Wisconsin
Source: Sci Rep. 2018 Aug 20;8:12440. doi: 10.1038/s41598-018-30988-4 (PMC6102245; doi:10.1038/s41598-018-30988-4)

A Bayesian state-space model using age-at-harvest data for estimating the population of black bears (*Ursus americanus*) in Wisconsin

Maximilian L. Allen, Andrew S. Norton, Glenn Stauffer, Nathan M. Roberts, Yanshi Luo, Qing Li, David MacFarland, and Timothy R. Van Deelen

Supplementary Material 5 (5a-5h). Visual comparisons of posterior distributions to prior distributions.

Supplementary Material 5a. Visual comparison of litter sizes (LS) for black bears of each age class (a, b, c & d) in Wisconsin. Solid and dashed lines, respectively, indicate posterior and prior distributions. Points indicate means and whiskers bound the 2.5th and 97.5th percentiles.

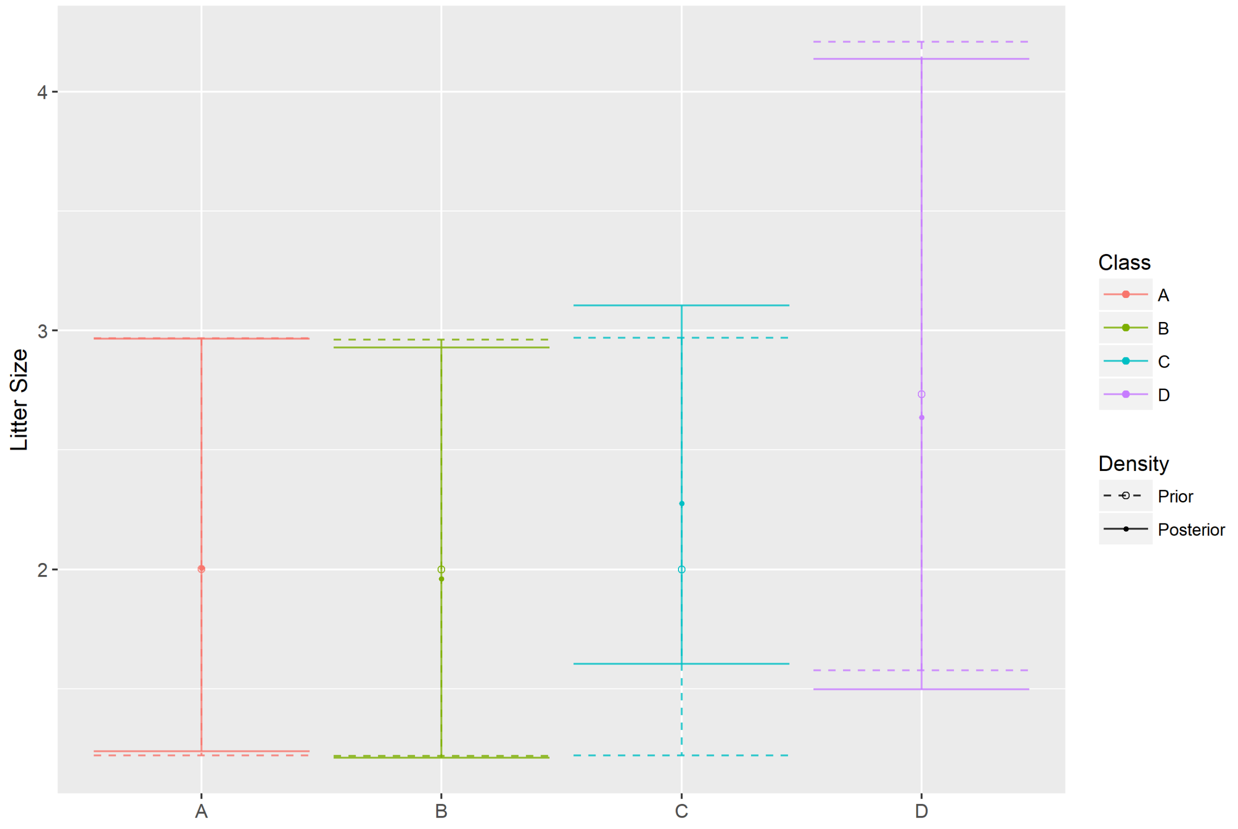

Supplementary Material 5b. Visual comparison of pregnancy rates (PR) for black bears of each age class (a, b, c & d) in Wisconsin. Solid and dashed lines, respectively, indicate posterior and prior distributions. Points indicate means and whiskers bound the 2.5th and 97.5th percentiles.

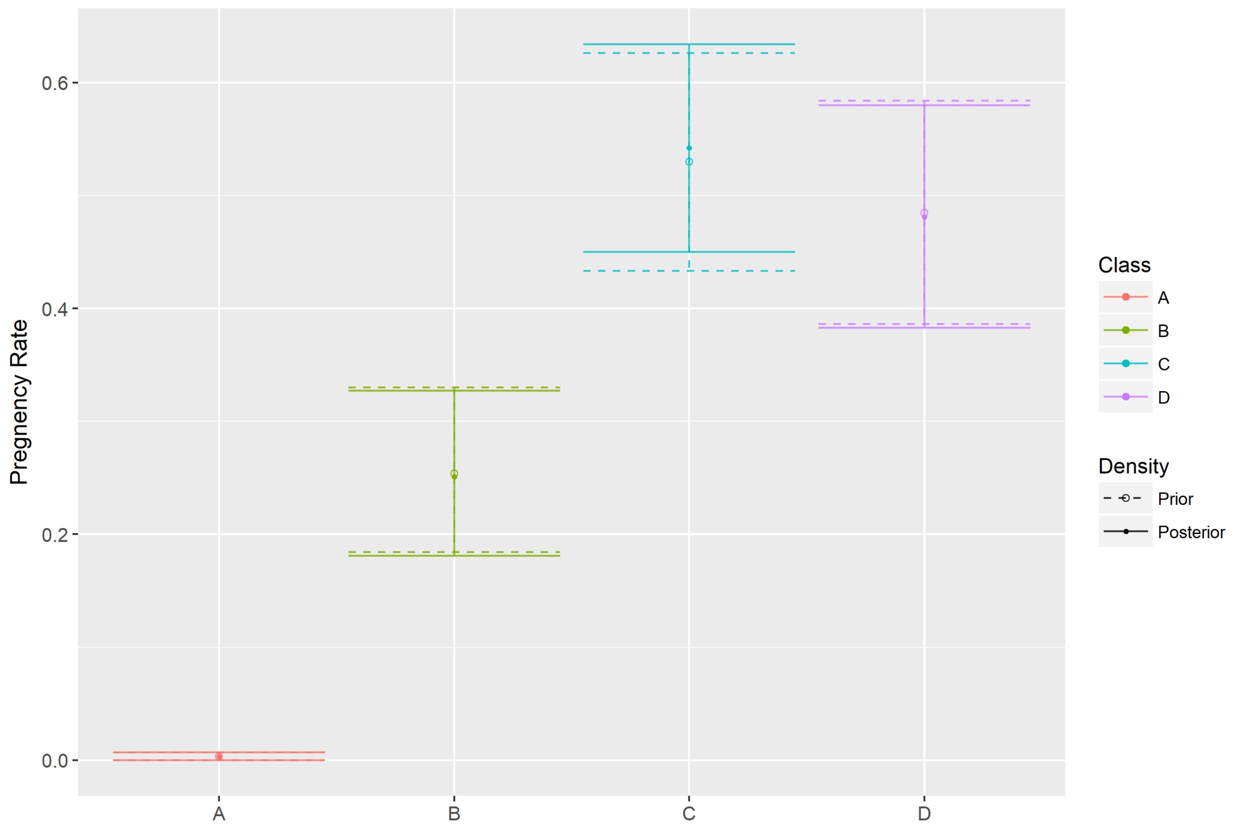

Supplementary Material 5c. Visual comparison of the sex proportion (SP) of cubs, shown as the percent of newborn cubs for each sex. Solid and dashed lines, respectively, indicate posterior and prior distributions. Points indicate means and whiskers bound the 2.5th and 97.5th percentiles.

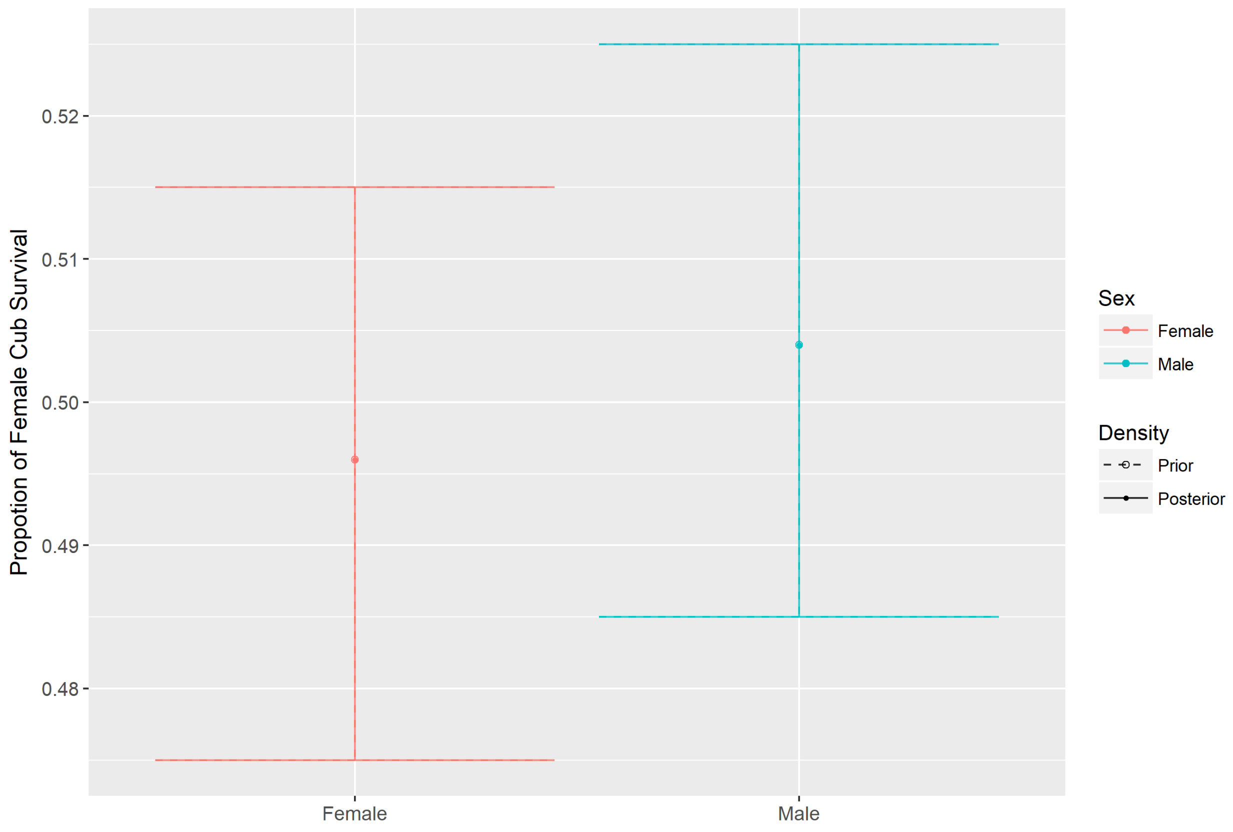

Supplementary Material 5d. Visual comparison of annual harvest season survival of black bears in each sex and age class in Wisconsin (2009-2016). Solid and dashed lines, respectively, indicate posterior and prior distributions. Points indicate means and whiskers bound the 2.5th and 97.5th percentiles.

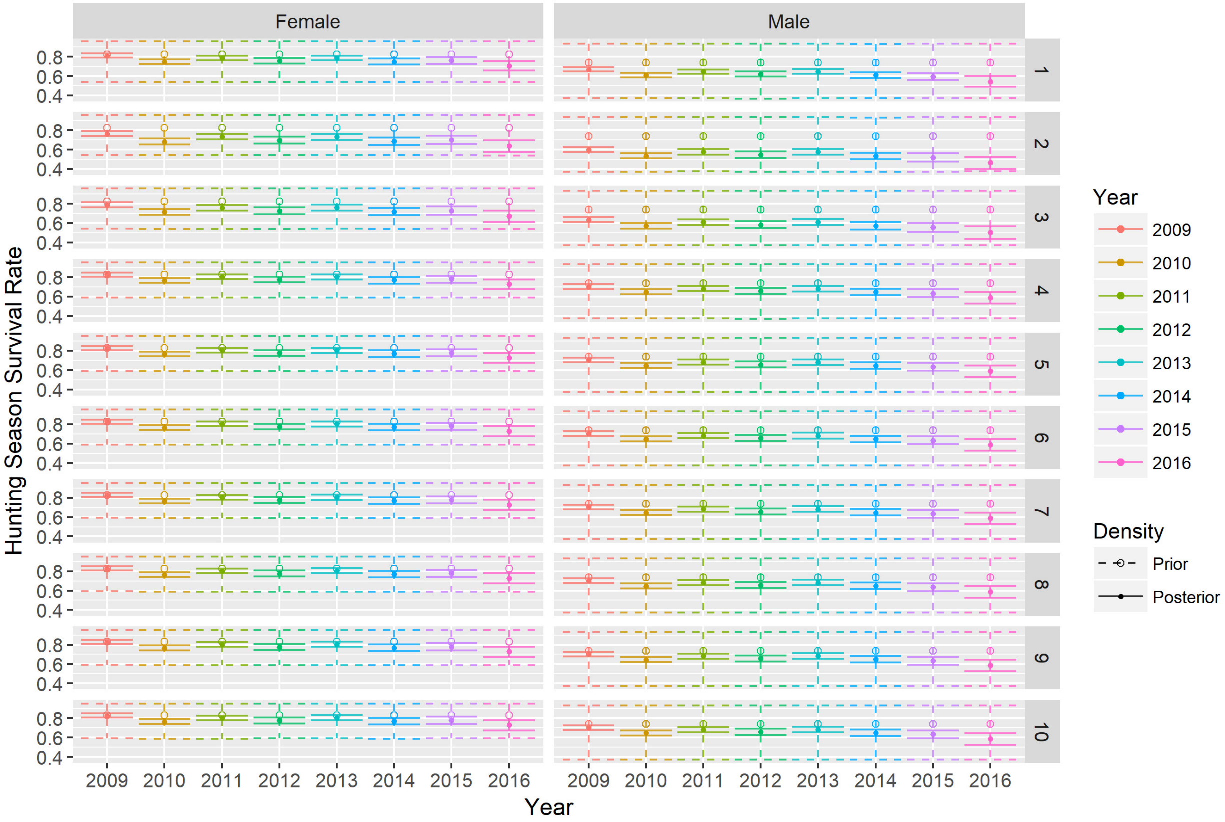

Supplementary Material 5e. Visual comparison of annual non-harvest season survival of black bears in Wisconsin (2009-2016). Solid and dashed lines, respectively, indicate posterior and prior distributions. Points indicate means and whiskers bound the 2.5th and 97.5th percentiles.

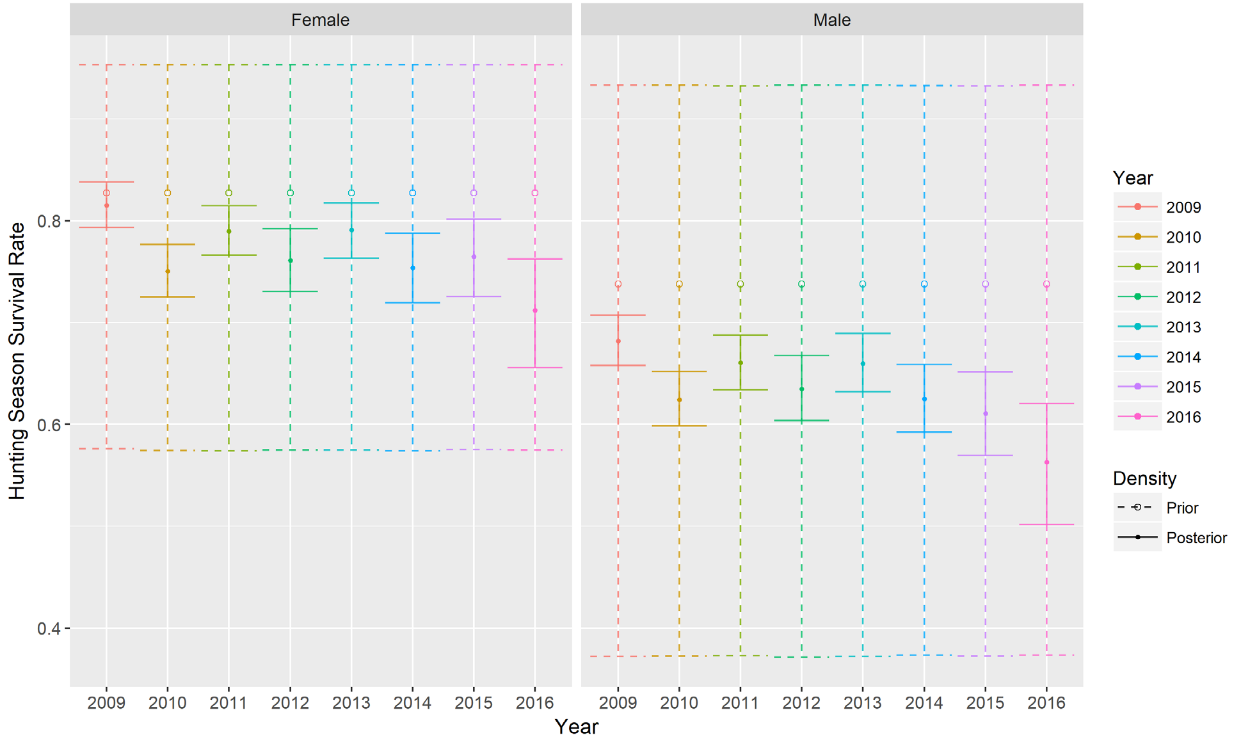

Supplementary Material 5f. Visual comparison of cub survival (CubS) for black bears in both periods (A = to 0.5 years, B = from 0.5 to 1.5 years) in Wisconsin. Solid and dashed lines, respectively, indicate posterior and prior distributions. Points indicate means and whiskers bound the 2.5th and 97.5th percentiles.

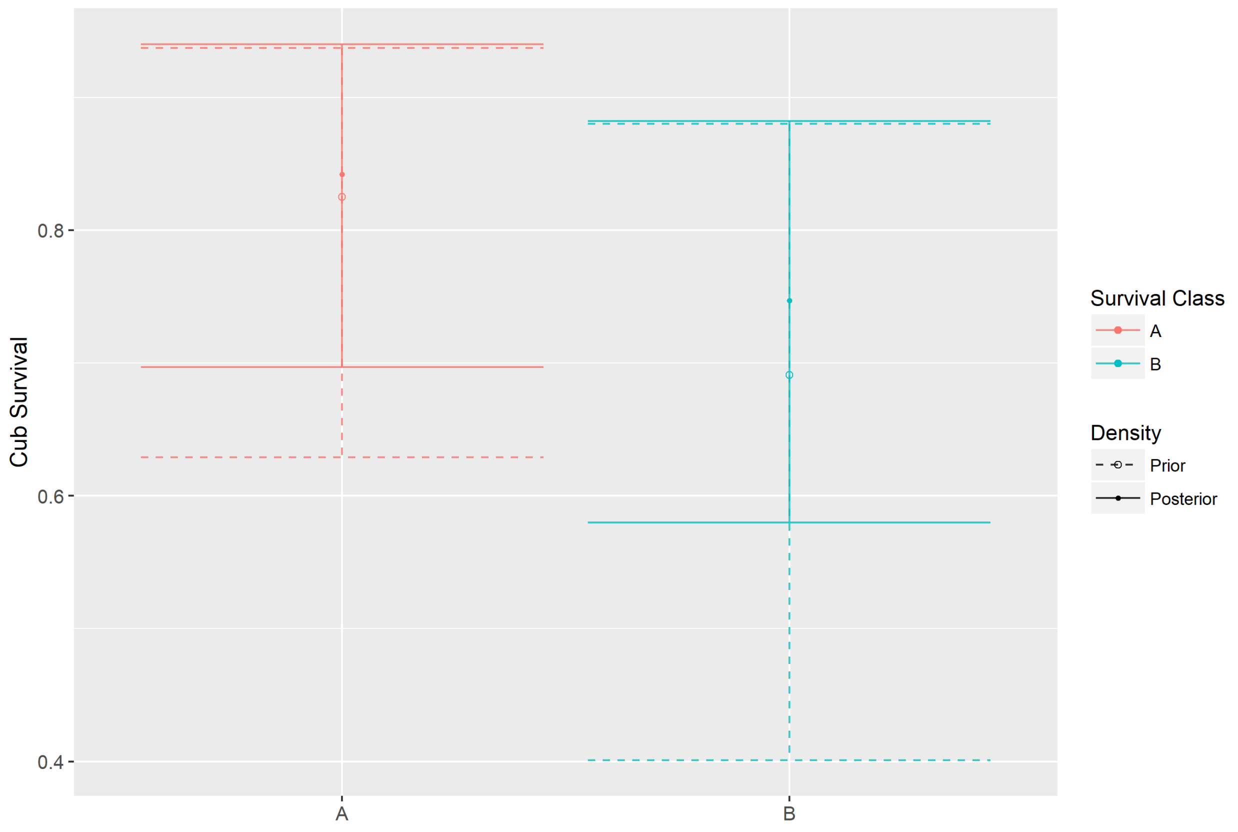

Supplementary Material 5g. Visual comparison of reporting rate for black bears of each sex in Wisconsin. Solid and dashed lines, respectively, indicate posterior and prior distributions. Points indicate means and whiskers bound the 2.5th and 97.5th percentiles.

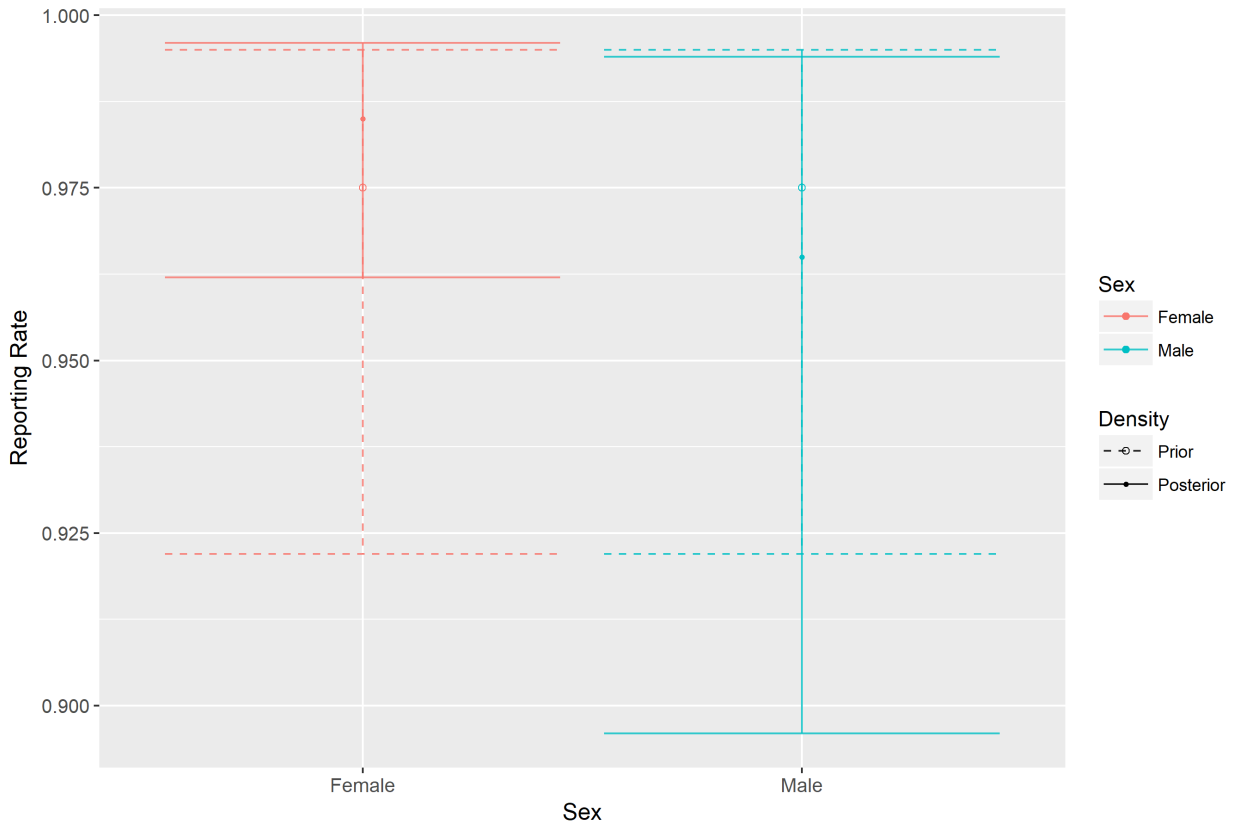

Supplementary Material 5h. Visual comparison of the initial population size of black bears in 2009 for each sex and age class in Wisconsin. Solid points indicate posterior means and whiskers bound the 2.5th and 97.5th percentiles. Open points indicate initial populations size values for each age class.

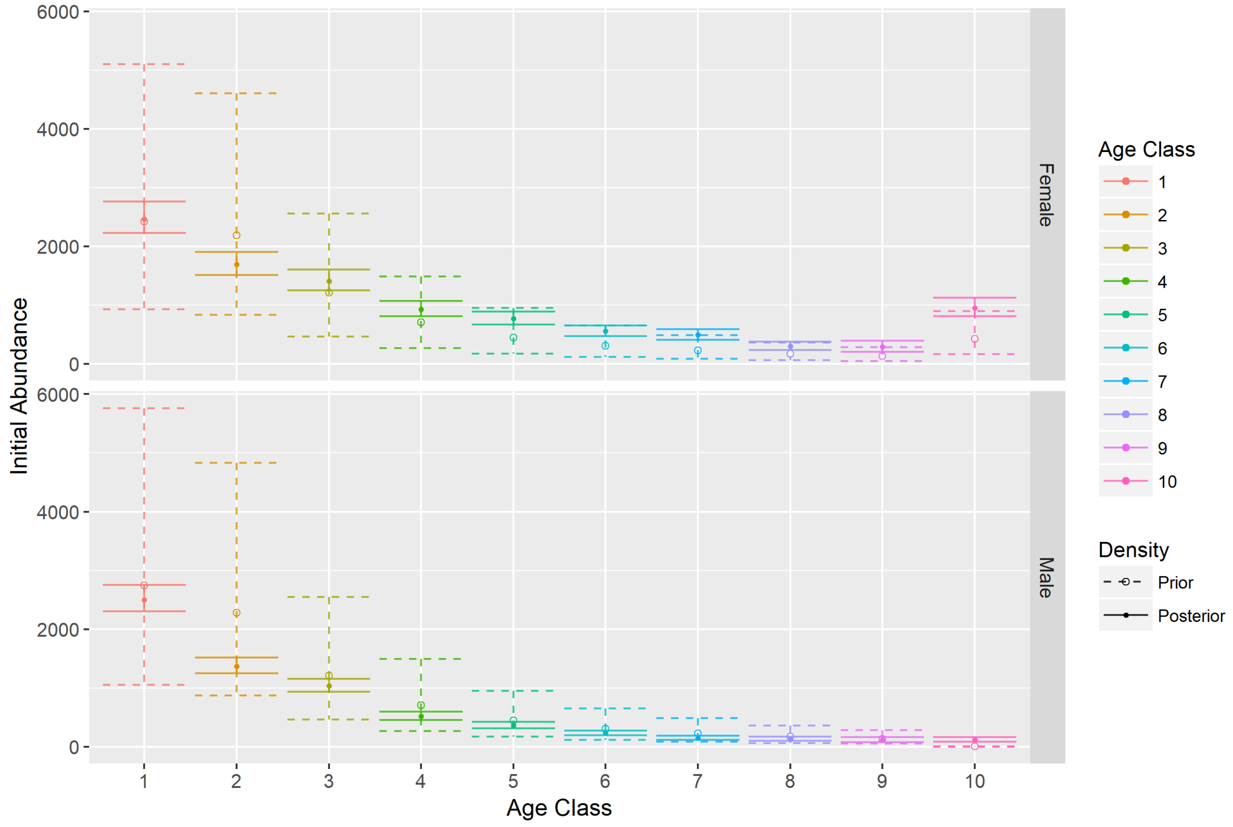

Supplement: Supplementary file 8 — Supplementary Material 5 [file 41598_2018_30988_MOESM8_ESM.pdf]
